# Role of primary aging hallmarks in Alzheimer´s disease

**DOI:** 10.7150/thno.79535

**Published:** 2023-01-01

**Authors:** Jin Zhao, Jisen Huai

**Affiliations:** The Second Affiliated Hospital of Xinxiang Medical University (Henan Mental Hospital), Xinxiang, 453000, China.; Institute of Psychiatry and Neuroscience, Xinxiang Medical University, Xinxiang, 453003, China.

**Keywords:** Aging, Alzheimer's disease, Epigenetics, Molecular neurobiology, Neurodegeneration, Oxidative stress

## Abstract

Alzheimer's disease (AD) is the most common neurodegenerative disease, which severely threatens the health of the elderly and causes significant economic and social burdens. The causes of AD are complex and include heritable but mostly aging-related factors. The primary aging hallmarks include genomic instability, telomere wear, epigenetic changes, and loss of protein stability, which play a dominant role in the aging process. Although AD is closely associated with the aging process, the underlying mechanisms involved in AD pathogenesis have not been well characterized. This review summarizes the available literature about primary aging hallmarks and their roles in AD pathogenesis. By analyzing published literature, we attempted to uncover the possible mechanisms of aberrant epigenetic markers with related enzymes, transcription factors, and loss of proteostasis in AD. In particular, the importance of oxidative stress-induced DNA methylation and DNA methylation-directed histone modifications and proteostasis are highlighted. A molecular network of gene regulatory elements that undergoes a dynamic change with age may underlie age-dependent AD pathogenesis, and can be used as a new drug target to treat AD.

## 1. Introduction

Dementia is an acquired loss of cognitive ability severe enough to interfere with daily living 1. Alzheimer's disease (AD) is the most prevalent form of dementia, affecting more than 50 million individuals worldwide 2. It is a progressive, amnestic, and fatal neurodegenerative disease characterized by the combined neuropathological burden of extracellular amyloid plaques and intracellular neurofibrillary tangles, two cornerstones of AD etiology 3-5. These pathological changes often occur selectively in the limbic and neocortical brain regions, especially in the entorhinal cortex layer II (ECII)**,** hippocampal CA1, and temporal and frontal lobes of the brain 6-8.

AD is a multifactorial disease with environmental (30%) and genetic (70%) causes. Environmental factors are usually associated with sporadic AD (SAD), while genetic factors are associated with familial AD (FAD) and SAD 9. Interestingly, FAD and SAD differ in age of onset 10-12. According to the age of onset, AD can be divided into two categories of early-onset AD (EOAD) and late-onset AD (LOAD) before or after the age of 65 10. In all AD cases, approximately 5% are EOAD and 95% are LOAD 11, indicating that most AD is caused by aging in concert with a complex interaction of genetic and environmental risk factors 13, 14.

AD, especially LOAD, is associated with aging and is characterized by selective neuronal vulnerability (SNV) 6, 7, 14, 15. However, the relationship between aging and SNV and the molecular basis of AD are not completely understood which need to be urgently elucidated 6, 16-18. Aging is the inevitable time-dependent decline in physiological organ integrity, leading to impaired function and increased vulnerability to death. It is characterized by nine tentative hallmarks grouped into three main categories: primary hallmarks (genomic instability, telomere attrition, epigenetic alterations, and loss of proteostasis), antagonistic hallmarks (deregulated nutrient sensing, altered mitochondrial function, and cellular senescence), and integrative hallmarks (stem cell exhaustion and altered intercellular communication) 19, 20. To date, the role of each aging hallmark in AD development remains unclear. This article will focus on the primary aging hallmarks as these are interconnected with other aging characteristics and are at the base of the hierarchical order of aging features 19, 20, and have been shown to be related to AD 21, 22. It is an attempt to improve our understanding of the pathological mechanisms of AD to find potential therapeutic approaches and diagnostic tools.

## 2. Genomic instability in AD

Recently, DNA damage has been shown to play a central role in aging and affects most aspects of the aging phenotype 23, 24, suggesting that DNA damage is a potentially unifying cause of aging 25. DNA damage markers, including double-strand breaks (DSBs), have also been found in brain regions of AD patients 26-30, indicating that DNA damage may be an important pathological cause of AD, particularly in LOAD cases 21, 31, 32.

It is conceivable that easily damaged regions in AD may be more active in normal physiological processes, including oxidative stress, neuronal activation, and gene transcription, causing DNA damage in neurons 33-36. However, to offset excessive DNA damage and prevent mutation, all organisms have evolved highly conserved DNA damage detection and repair mechanisms 37-41. How the threshold of this homeostatic DNA damage and repair is disrupted has yet to be completely understood 42. It has been proposed that the balance between DNA damage and repair processes is disrupted mainly by DNA repair becoming less efficient with age, finally causing genomic instability, and triggering cell death signalling cascades 23, 43, 44. This is consistent with the idea that DNA damage accumulation during the lifetime in differentiated cells such as neurons is mainly attributed to insufficient DNA repair 45-47. However, an age-dependent increase in DNA damaging factors such as reactive oxygen species (ROS) has also been reported, likely inducing more DNA damage 43, 48.

The human brain accounts for approximately 2% of our body mass, but it uses 20% of the total oxygen supply consumed by the whole body 49, 50. It requires a continuous supply of energy in the form of ATP, produced by oxidative phosphorylation in mitochondria with ROS as the by-product 51. Among the three main types of ROS, superoxide radicals (•O_2_^-^), hydrogen peroxide (H_2_O_2_), and the hydroxyl radical (•OH), •O_2_^-^ is the proximal mitochondrial ROS, which can be converted to H_2_O_2_ by superoxide dismutase (SOD) and then to •OH by Fenton´s reaction of H_2_O_2_ with Fe^2+^ 52-54. •OH is by far the most active form of ROS and is considered the main DNA damaging factor in neurons and can cause altered bases, abasic sites, and single- and double-strand breaks 55-57. Excessive ROS can lead to approximately 100 different oxidative base damage and 2-deoxyribose modifications most likely to occur in neuronal cells 55, 58. Oxidative DNA damage is notably related to human diseases 48, 59 and is the most significant DNA lesion affecting the progression of AD 60.

It has been shown that 8-oxo-2-deoxyguanosine (oxo8dG), a marker of oxidative DNA damage, accumulates with age in nuclear DNA in all tissues and strains of rodents and is associated with AD brain pathology 57, 61, 62. However, the accumulation of oxyradical-associated DNA damage in different brain regions is not uniform 46, 63, 64. The vulnerable areas in the brains of AD patients appear to be more sensitive to DNA damage insult and are also subjected to DNA repair deficiency with age. Although it is difficult to determine which of the two processes plays a more important role in AD development, DNA damage and repair deficiency contribute to the genomic instability, thereby causing systematic regional vulnerability of AD 33, 65, 66. DNA damage may reduce the expression of selectively vulnerable genes involved in learning, memory, and neuronal survival, initiating a program of brain aging that starts early in adult life 33. Genomic instability affects the expression of the genes linked to mitochondrial and metabolic dysfunction, altered proteostasis, and inflammation, which are centrally involved in aging, and supports the notion that DNA damage could be the root of aging and AD 23, 67. Therefore, targeting DNA damage or other aging-related hallmarks provides a rationale for developing interventions to combat age-related diseases, including AD 25.

Neurons are post-mitotic cells vulnerable to oxidative damage comparable to dividing cells during aging 68. However, neurons from different AD brain regions show significantly different vulnerability, are equipped with different antioxidants, and have different abilities to scavenge oxidized DNA groups, which play a decisive role in the SNV of AD 63, 69. Therefore, the cell-autonomous mechanism is thought to be the primary driver responsible for initiating SNV in AD 16. However, this notion is challenged by other findings. For example, although the regional basal levels of DNA damage inversely correlate with the regional capacity to remove oxo8dG from DNA 63, it has been reported that the age-related increase in oxo8dG in the nuclear DNA of aging mice is not due to a decrease in the ability to eliminate oxo8dG damage. Instead, the increase in oxo8dG levels seems to be caused by an age-related increase in the sensitivity of the tissues to oxidative stress 61, 70. Intriguingly, the sensitivity of neurons to oxidative stress also depends on their surrounding environment. Neurons from different regions may have distinct surrounding cells and tissues, such as astrocytes, microglia, and vasculature, which can secrete other antioxidants or oxidants, thereby inhibiting or promoting ROS damage 71-78. These observations suggest that the surrounding environment may play a dominant role in maintaining DNA integrity and the survival of neurons. Consistent with this hypothesis, a transplantation experiment showed that the lifespan of neurons was not limited by the maximum lifespan of the donor organism but was influenced when transplanted in a longer-living host 79. Although both oxidative DNA damage and repair deficiency contribute to genomic instability and are involved in the neurodegenerative process of the AD brain 80, 81, oxidative DNA damage, which increases with age, appears to play a leading role. However, it still needs to be clarified whether neuronal DNA damage is caused mainly by autonomous or nonautonomous mechanisms and how the critical point for maintaining neuron redox balance is impaired and causes cell death and AD onset.

## 3. Telomere attrition in AD

The telomere is the protective end cap of the chromosome, a nucleoprotein complex composed of tandem TTAGGG DNA repeats and associated protein complexes known as shelterin 82, 83. The shelterin complex consists of six associated proteins: telomeric repeat binding factor 1 and 2 (TRF1/2), TRF2 interacting protein (RAP1), TRF1-interacting nuclear factor 2 (TIN2), adrenocortical dysplasia protein homolog (TPP1), and protection of telomeres 1 (POT1) 83, 84. The shelterin complex is believed to stabilize a lariat-like structure of the chromosomal end, the telomere loop (t-loop), to prevent telomeres from inadvertently activating DNA damage signaling and DSB repair pathways 85-87.

Telomeres shorten with age, and approximately 50 nucleotides are lost during each cell cycle 88. Although neurons are post-mitotic cells, and their telomeres are not expected to be shortened through the division mechanism, they may still accumulate irreparable DNA damage, resulting in a senescent cell type or even apoptosis 89-92. In contrast, neural stem cells (NSCs) are proliferative and affected by aging 93. Telomere maintenance is crucial for NSC viability and self-renewal potential, while telomere shortening may cause cognitive impairment and psychiatric disorders 94, 95. Nonneuronal brain cells such as NSCs and astrocytes divide more slowly than cells from other tissues, and the telomere attrition rate is slower than other cells 94. Leukocyte telomere length (LTL) has been widely used as a surrogate marker for central nervous system telomere length (TL) 96, and an association between accelerated LTL shortening and the incidence of AD has been demonstrated 94, 97-99 despite several negative reports 100-102. Since telomeres are the protective chromosomal end caps, their dysfunction may lead to genomic instability 103, 104. For example, altered telomere chromatin structure has been linked to a defective DNA damage response 103. Since genomic instability plays a key role in the pathogenesis of AD, it is not surprising that telomere attrition is associated with the incidence of AD. Deficits in telomere-associated enzymes such as telomerase and DNA-PKcs render neurons vulnerable to adverse conditions related to AD. In contrast, telomerase-increasing compounds protect hippocampal neurons from Aβ42 toxicity by enhancing the expression of neurotrophins and plasticity-related genes 105-107, confirming that telomere attrition may play an important role in AD pathogenesis.

Remarkably, the telomeres of hippocampal neurons become shorter in AD, and much evidence supports its correlation with elevated levels of oxidative stress 102, 108. Interestingly, telomeres are not only favoured by oxidative attack due to their unique sequence but also less efficiently repaired when compared to non-telomeric damage 109-112. Furthermore, genomic instability can adversely affect telomere integrity 113, 114. For example, the DNA damage response can cause telomere erosion 113. These results suggest that oxidative stress may cause SNV of AD through both non-telomeric and telomeric DNA damage in a synergistic manner and are consistent with the findings that oxidative stress plays a central role in the pathogenesis of AD 115, 116. Furthermore, in mature human hippocampal neurons and activated microglia, telomerase reverse transcriptase (TERT) also plays a protective role against oxidative damage independent of its canonical function of telomere maintenance 117, 118. In line with this finding, TERT protein is increased in mitochondria of AD hippocampal CA1 neurons compared to healthy controls 117, probably due to the compensatory effect of cells on oxidative stress in AD. However, overexpression of TERT or boosting its activity through compounds cannot prevent AD, although it has antioxidative and autophagy-promoting effects 89, 119, 120. Collectively, telomeres form a special structure at the end of the chromosome to protect the integrity of the genome in canonical and noncanonical ways and are very important for the survival of neural cells. However, the telomere is very sensitive to oxidative stress and vulnerable to damage. Therefore, elevated free radical pressure may lead to telomere wear with increasing age, exacerbating the related genomic instability, and contributing to AD pathogenesis.

## 4. Epigenetic alterations in AD

Epigenetics focuses on the underlying mechanism of gene expression without changes in DNA sequences. The most studied epigenetic signatures are DNA methylation, histone modification, and noncoding RNAs (ncRNAs) 121. Epigenetic mechanisms directly contribute to aging and aging-related diseases 122. Recently, neuro-epigenetics has emerged as an important field that explores how reversible modifications can change gene expression to control behavior and cognitive abilities 123. Accumulating findings have shown that epigenetic machinery plays a key role in maintaining genome integrity and regulating gene expression 124, and its dysfunction is closely related to AD pathogenesis 125.

### 4.1. DNA methylation

DNA methylation refers to the attachment of a methyl group to the DNA chain. While methylation is catalyzed by DNA methyltransferases (DNMTs), including DNMT1 (the primary maintenance DNA methyltransferase), DNMT3A, DNMT3B, and DNMT3L 126, 127, demethylation is mainly performed by ten-eleven translocation (TET) family enzymes 128, 129. DNA methylation primarily occurs on the 5^th^ carbon of a cytosine residue (5mC) at CpG and non-CpG (CpA, CpC and CpT) dinucleotides 130. In vertebrates, CpG dinucleotides are predominantly methylated in all tissues, with approximately 80% of all CpG sites containing 5mC 131-133. In contrast, non-CpG methylation (mCpHs, H = A/C/T) is usually restricted to specific cell types, such as pluripotent stem cells, oocytes, neurons, and glial cells, and plays a critical regulatory role in cognitive function 127, 134. CpH methylation can be up to 25% of all CpH sites in adult mouse dentate neurons, and it is generated de novo during neuronal maturation and requires DNMT3A for active maintenance in post-mitotic neurons 132.

**Figure 1:**
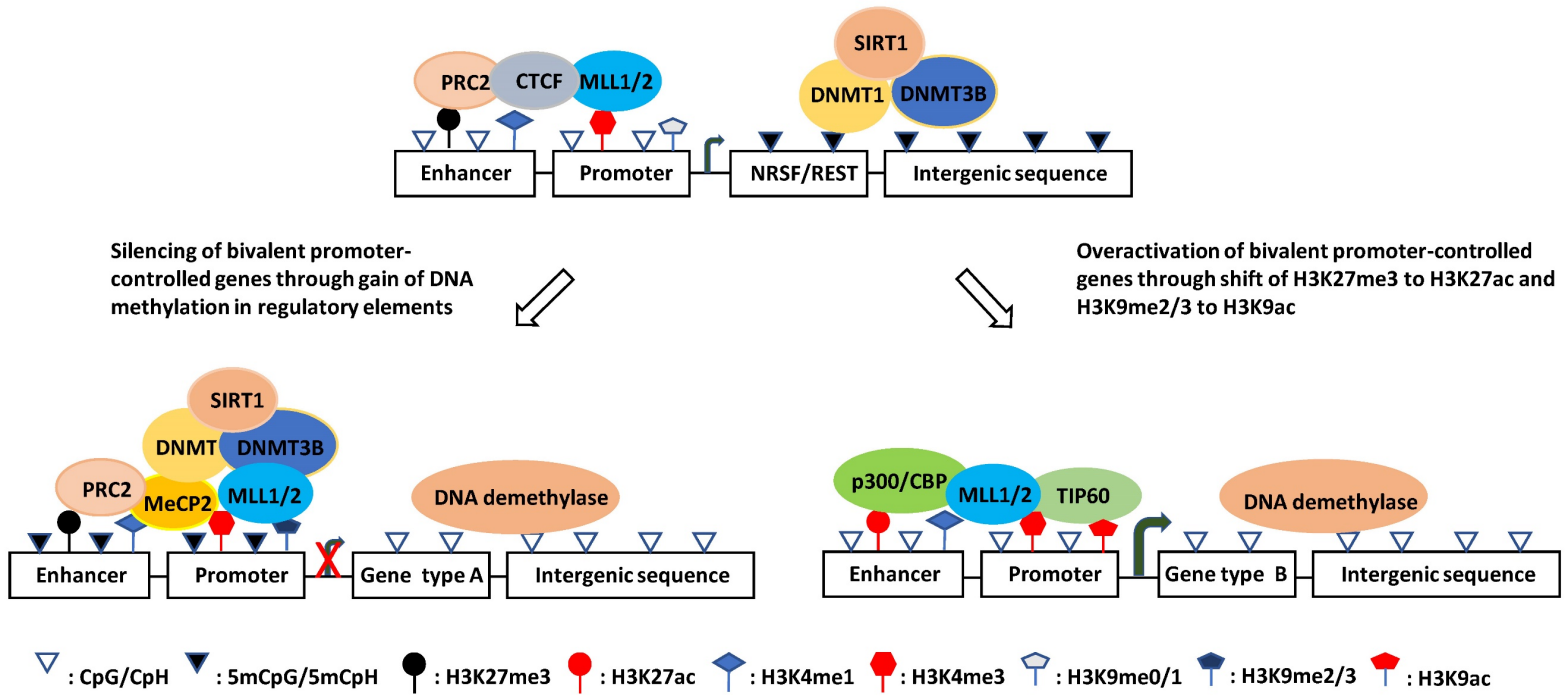
** Schematic illustration of the hypothesized mechanisms of aberrant gene expression in AD.** (left) Downregulation mechanism: DNMTs shift from non-CGI regions to CGI regulatory elements under oxidative stress and catalyze DNA methylation at the damage sites, which recruits MeCP2 and help DNMTs catalyze more DNA methylation thereby resulting in the target gene silencing. (Right) Upregulation mechanism: DNA damage repair induces co-occurence of H3K9ac and/or H3K27ac with H3K4me3, which prevents DNA methylation and represses H3K9me2 inhibitory effect on transcription to overactivate the genes.

Intriguingly, although genome-wide CpGs are highly methylated, CpG islands (CGIs) are paradoxically unmethylated in most cases 127, 135, 136. These CGIs are usually cis-regulatory elements, including more than 50% of mammalian gene promoters 133, 137-139. They are high-intensity CpG promoters (HCPs) regulating housekeeping genes, developmental regulator genes, and a proportion of tissue-specific genes 140, 141. By contrast, the remaining over 40% of human gene promoters are low-intensity CpG promoters (LCPs, also known as non-CGI promoters: NCPs), which are differentially methylated and are more prone to hypermethylation under stress 138, 142-144. Recently, it was reported that methylated CpHs (mCpHs) and CpGs (mCpGs) in the brain increase with age 145-147. Enhancers with hypermethylated CpHs are associated with genes functionally enriched in immune responses, and some of the genes are related to neuroinflammation and degeneration 145, 148, 149. In general, DNA methylation in enhancers and promoters negatively regulates gene expression, whereas gene body methylation likely increases transcriptional activity (Fig. 1) 149-152. However, some studies have shown that hypomethylation in the promoter region is associated with the downregulation of gene expression 153.

Aberrant DNA methylation has been linked to many AD susceptibility genes, including amyloid precursor protein (APP), β- and γ-secretases 153-156, apolipoprotein E 157, 158, Triggering receptor expressed on myeloid cells 2 (TREM2) 159, hTERT 160, cAMP responsive element binding protein (CREB)-regulated transcription coactivator 1 (CRTC1) 161, brain-derived neurotrophic factor (BDNF) 162, thromboxane A2 receptor (TBXA2R, related to CREB activation), sorbin and SH3 domain-containing 3 (SORBS3, related to synapse formation), and spectrin beta 4 (SPTBN4, related to axon initial segment) 163. Epigenome-wide association studies (EWAS) have uncovered more genes with altered DNA methylation levels in the hippocampus, entorhinal cortex, dorsolateral prefrontal cortex, and cerebellum of both early and late AD patients as compared with controls 164-167. Interestingly, many of these genes were previously shown to be AD susceptibility genes or connected to the AD susceptibility network and participate in different pathological processes of neurons or nonneuronal cells, such as amyloid pathology (APP, ABCA7, SERPINF1 and 2), tau pathology (BIN1), inflammation (ANK1, RHBDF2, IL-1β, and IL-6), protein dyshomeostasis (RPL13 and HOXA3), calcium dyshomeostasis (S100B), and cellular skeleton defects (MAP2 and MCF2L) 164-167.

A genome-wide DNA methylation analysis of hippocampal or superior temporal gyrus samples from a cohort of AD patients and controls used Illumina 450K methylation arrays. The study showed that differentially methylated positions (DMPs) of AD patients were enriched in poised promoters (bivalent promoters) marked by H3K27me3 and H3K4me3, which are not generally maintained in committed neural cells but participate in neurodevelopment and neurogenesis (Fig. 1) 168, 169. This is consistent with previous findings that epigenetic mechanisms are critical to adult hippocampal neurogenesis and are relevant to AD etiology 170-173. It is conceivable that the impaired generation of neurons from NSCs will exacerbate the loss of neurons causing learning and memory deficits 174, 175.

Although the AD susceptible genes are regulated by DNA methylation, the mechanism for the aberrant methylation patterns is not fully understood. The primary point for DNA methylation in mammalian genomes is cytosine in CpG dinucleotides, which are highly enriched in CGIs, but paradoxically, CGIs are usually unmethylated 127. It was proposed that CGI promoters (HCPs), which are transcriptionally active at totipotent stages of development, can act as origins of DNA replication; thus, these CGIs can exclude methylation due to occupancy with the molecules that initiate DNA replication 176, and the methylation-free form of the promoter is transmitted to all somatic cells 177. Although most of these CGI promoters remain transcriptionally active (high expression HCPs), others become silenced by recruited polycomb repressive complex 2 (PRC2), which catalyzes H3K27me3 178-180, or become poised with the coexistence of H3K27me3 and an activating histone marker H3K4me3 (poised HCPs) 181, 182. Under stress conditions, the PRC2 catalytic subunit enhancer of zeste homolog 2 (EZH2) can recruit DNMTs, catalyzing de novo DNA methylation 183-185. Critically, DNA methylation is enriched in poised promoters of AD susceptibility genes 168, 169. Given that H3K4me3 protects against DNA methylation 186, 187, whereas H3K27me3 promotes DNA methylation, the ratio of H3K4me3/H3K27me3 appears to play a key role in determining the DNA methylation state at the CGI promoters. This is consistent with the finding that DNA methylation at these CGIs can be coordinated by H3K27me3 188-190.

O´Hagan et al. found that under oxidative stress, DNMT1/DNMT3B, together with SIRT1, move from non-GC-rich regions to CGIs, forming a complex with two subunits of the PRC2 complex (EZH2 and EED2), and cause local DNA methylation 191. Also, DNA methylation under chronic oxidative stress occurs only on low expression (poised HCPs) but not high expression CGI promoters (Fig. 1) 191. The reason why highly expressed HCPs are protected from methylation is poorly understood. As mentioned previously, the molecules responsible for DNA replication might occupy the sites and exclude methylation of high-expression CGI promoters 176, 177. It is also possible that proteins containing a CXXC zinc finger domain and its resultant H3K4me3 inhibit the activity of the de novo DNA methyltransferase DNMT3a, thus preventing methylation at these sites 144, 192. In addition, HCPs with high expression contain high CpG intensity and are less occupied and can, therefore, produce more oxo8dG and 5hmC under oxidative stress. However, oxo8dG can induce DNA hypomethylation by inhibiting DNA methylation at nearby cytosine bases, and 5hmC can cause hypomethylation by activating DNA demethylation 193. Therefore, so far, it is unclear which of these mechanisms plays a leading role in protecting highly expressed HCPs from methylation.

Notably, whilst the high-expression HCPs are protected from methylation, other mechanisms can still silence the genes. Either the specific oxo8dG base damage or the base excision repair (BER) pathway that repairs this type of damage can recruit members of the gene silencing complex to these promoters 191. This is consistent with previous findings that oxo8dG is associated with the pathological process of the AD brain 57, 61, 62. Additionally, 5hmC in the inferior parietal lobe, hippocampus, parahippocampal gyrus, and cerebellum of patients with mild cognitive impairment (MCI) and preclinical Alzheimer's disease (PCAD) has been shown to be significantly increased 194. These findings may explain why housekeepers such as glyceraldehyde-3-phosphate dehydrogenase (GAPDH) and β-actin (controlled by high expression CGI promoters) have a lower overall expression in AD cases than in controls 195. Interestingly, many defective DNA repair genes in AD are not methylated in their promoters 196, 197, although DNA damage response factors, such as p53, poly(ADP-ribose) polymerase 1 (PARP-1) and Growth arrest and DNA damage-inducible alpha (GADD45a), have been shown to stimulate DNA methylation 198, 199. Whether they are silenced by oxo8dG/BER-recruited silencing complexes is although likely but not known. In support of this notion, it has been reported that alteration of the expression of repair genes is controlled by aberrant transcriptional and epigenetic factors in addition to DNA methylation/demethylation and gene mutations 198.

In summary, DNA methylation is involved in both aging and AD. However, many de novo methylations of initially unmethylated sites are AD-specific and play an important role in AD pathogenesis. Although the detailed mechanism for aberrant DNA methylation patterns is unclear, there is evidence showing that oxidative DNA damage plays a key role. Since DNA methylation and damage response are closely related, it is tempting to propose that AD-specific methylation patterns derive from and reflect stronger oxidative damage, requiring extensive repair and therefore generating additional errors. Although the hypo- or unmethylated CGI promoters may become hypermethylated or remain unchanged under chronic oxidative stress, gene silencing complexes may form on both low- and high-expression HCPs. These complexes can coordinate different epigenetic markers and thereby determine gene transcription 191, 200-205. Various DNA methylation states on promoters may form distinct patterns of other epigenetic modifications 206-208. In line with this notion, it has been reported that histone modifications occurring on HCPs and LCPs are distinct and active LCPs require H3K4me3 and H3K79me1, while active HCPs require H3K27ac and H4K20me1 209, 210. In the next section, we summarize new findings of histone modifications and their interaction with DNA methylation.

### 4.2. Histone modification

Histone modification is another pivotal form of epigenetic regulation. Histones are covalently modified, altering the intrinsic chromatin structure, and regulating gene expression 211, 212. In eukaryotes, the core structure of chromatin is a nucleosome, composed of a histone octamer formed by two copies of each of the four histones (H2A, H2B, H3, and H4) and 147 base pairs of DNA wrapped around the octamer 213. Various posttranslational modifications occur at histones, the most common being methylation, acetylation, phosphorylation, and ubiquitination 121, 214. Histone modification usually occurs on gene promoters and enhancers, with methylation and acetylation at positively charged lysine (K) and arginine (R) residues being the most prevalent 211, 215, 216. Histone modification is dynamic and reversible and is controlled by both writers (e.g., histone acetyltransferases (HATs) or histone methyltransferases (HMTs)) and erasers (e.g., histone deacetylases (HDACs) or histone demethylases (HDMs)) required for proper in-printing and off-printing of genes 217. The imbalance between writers and erasers, resulting in epigenetic marker disparity and transcription dysfunction, is associated with many brain disorders 218-221. Accumulating evidence has shown that histone methylation and acetylation dysregulation is associated with AD pathology even in its early stage 222-226.

#### 4.2.1. Histone acetylation variation in the AD brain

Dysfunction of HDACs (HDAC1, HDAC2, HDAC3, and HDAC6) has been reported to be involved in cognitive impairment, a debilitating feature of many neurodegenerative disorders, including AD 227-233. At late AD stages, HDAC1 and HDAC2 were shown to be decreased in the prefrontal cortex (PFC) and hippocampus of AD patients 234, 235. Lu et al. found that H3K9ac was significantly upregulated in early AD PFC neurons, whereas it was globally downregulated in normal aging PFC neurons 236. In addition, Nativio et al. reported that H3K9ac was also enriched in the AD temporal lobe together with H3K27ac 237, but no information was available on the level of H3K9ac in the AD hippocampus. However, Tat-interacting 60 kDa protein (TIP60/KAT5, a HAT for expression of H3K9ac, H3K14ac, and H2AK5ac) was significantly reduced in both neurons and glial cells and largely absent from nuclei of neurons in the AD hippocampus 238, where TIP60 and GCN5/KAT2A were usually most strongly expressed 239-241, suggesting that H3K9ac and other acetylated histones catalyzed by TIP60, were decreased in the AD hippocampus. Since many cognition-related genes are regulated by H3K9ac 242, TIP60 reduction in the hippocampus might downregulate these cognition-related genes, which would be upregulated by increased H3K9ac in the PFC and temporal lobe. However, the genes involved in apoptosis (BAX and DAXX), Aβ production (presenilin-2), and neurotransmission (SST and CALB1) are also upregulated in the PFC and temporal lobe 236. Thus, both H3K9ac downregulation in the hippocampus and upregulation in the PFC and temporal lobe of the AD brain might be detrimental.

An increase in genome-wide levels of H3K9ac and H3K27ac in a fly model of AD exacerbated Aβ42-driven neurodegeneration 237. Enrichment of H3K9ac and H3K27ac on the angiopoietin-like protein 4 (ANGPTL4) gene promoter has also been shown to be involved in inflammation, vascular permeability, and metabolic dyshomeostasis 241, 243, and the expression of ANGPTL4 in brain glial cells was stimulated by hypoxia 244, 245, suggesting that enrichment of H3K9ac and/or H3K27ac in the brain may be a typical response to hypoxia 246. However, in contrast to H3K9ac and H3K27ac, H3K18ac, H3K23ac and H4K16ac were significantly downregulated or lost in the temporal lobe of the AD brain 247, 248. In AD, hippocampal CA1 H3K12ac was downregulated 249, although it was significantly elevated in peripheral monocytes 250. Collectively, both upregulation and downregulation of these histone markers (H3K9ac, H3K27ac, and others) (Table 1) may cause cognition defects. Furthermore, H3K9ac upregulation in the PFC and temporal lobe and possibly the downregulation in the hippocampus plays a key role in AD pathogenesis, suggesting that H3K9ac might be a promising drug target for AD therapy.

Of note, H3K9ac has been shown to co-localize with H3K14ac on the previously described poised promoters harbouring H3K4me3 and H3K27me3 209, 251, 252. The genes controlled by poised promoters are usually expressed at basal levels due to the coexistence of H3K27me3 and H3K4me3 181. Since H3K9ac is an active gene marker, and both H3K9ac and H3K14ac are involved in DNA damage repair 253, 254, their co-occurrence on poised promoters suggests that the expression level of these genes can be increased in response to DNA damage 181. They are primed by H3K14ac, which is catalyzed by p300/CREB binding protein (CBP, a HAT that can also switch H3K27me3 to H3K27ac downstream of PHF8/KDM7B/JMJD-1.2), p300/CBP-associated factor (GCN5/PCAF) and/or MYST3, and then activated by H3K9ac, which is mainly catalyzed by GCN5/PCAF and/or TIP60 251. Consistent with this finding, H3K18ac, H3K23ac, and H4K16ac were found to be involved in the DNA damage response 255-257. While H3K18ac enrichment promotes the transcriptional expression of nucleotide excision repair (NER)-related genes and inhibits DNA damage 255, H3K23ac is coupled to H3K14ac, which is known to facilitate DNA repair in a positioned nucleosome by stabilizing the binding of the chromatin remodeler 254, 256. Also, H4K16ac plays an important role in DNA damage repair by modulating the recruitment of the DNA damage repair protein Mediator of DNA damage checkpoint 1 (MDC1) 257. Critically, H3K18ac, H3K23ac, and H4K16ac occur mainly on promoters and/or enhancers 248, 256, 258-261 and may affect the presence of H3K9ac and H3K27ac and/or cooperate with them to determine whether the gene is activated or repressed in response to DNA damage. However, although it is known that H3K9ac is upregulated in the PFC and temporal lobe, while H3K18ac, H3K23ac, and H4K16ac are downregulated or lost in the temporal lobe of the AD brain 247, 248, information on these factors throughout the AD-affected brain regions is still needed.

Moreover, the expression of H3K9ac is normally inhibited by the level of repressor element 1 (RE1)-silencing transcription/neuron-restrictive silencer factor (REST/NRSF) 236. REST is a gene-silencing transcription factor (a Krüppel type zinc finger protein), which can bind to thousands of sites in the human genome and repress a large array of coding and noncoding neuron-specific genes 262, 263 by interacting with many chromatin-modifying enzymes, including HDACs (e.g., HDAC1,2), HMTs (e.g., G9a), HDMs (e.g., lysine-specific demethylase 1 (LSD1/KDM1A)), and methyl CpG binding protein 2 (MeCP2) 263-265. However, REST was found to be absent from the PFC and hippocampus only at late stages of AD 236 suggesting the existence of a mechanism compromising the inhibitory effect of REST on H3K9ac at the early stage of AD. Although the potential mechanism is currently unclear, it is conceivable that chromatin-modifying enzymes associated with REST play a role. This notion is supported by the observation that TIP60 and plant homeodomain finger protein 8 (PHF8/KDM7B/JMJD-1.2, an HDM for demethylation of H3K9me1/2 and H3K27me2) could form a REST-interacting complex and increase the local H3K9ac/H3K9me2 ratios 266-270. Interestingly, TIP60 is also the core component of the DNA damage repair machinery and can acetylate ATM at damaged sites to regulate DNA repair signals 266.

In summary, histone acetylation markers, especially H3K9ac and H3K27ac, can become aberrant in AD (Table 1) and may lead to abnormal activation or overactivation of the affected genes, which may cause cell death. However, the aberrant expression of H3K9ac and H3K27ac in other regions of the AD brain is unclear, and the underlying mechanism that causes aberrant expression of H3K9ac is just beginning to be understood. Although DNA damage repair is involved and TIP60 may play an important role, details are still missing. For example, it is unclear whether TIP60 acts on histone modifications other than H3K9ac in response to DNA damage and the disparity between the levels of these markers is related to disease stages. Answering these questions will help develop a specific approach to downregulate H3K9ac for AD treatment.

#### 4.2.2. Histone methylation variation in the AD brain

According to previous reports, H3K9me2 in the occipital cortex and PFC is significantly elevated at the late stage of AD 221, 271 and downregulated in the hippocampal CA1 (Table 1) 249. H3K4me3, associated with memory formation 272, is also elevated in the AD PFC and superior and medial temporal gyrus but is downregulated in the AD entorhinal cortex and hippocampus (Table 1) 169, 273-275. Interestingly, gene-repressive H3K9me2 and gene-activating H3K4me3 markers are concomitantly and globally upregulated in the AD PFC and downregulated in the AD hippocampus. This observation indicates that gene activation and inhibition are active in the AD PFC, reflecting the compensatory state of gene expression. However, in the AD hippocampus, both gene activation and inhibition are inactive, indicating the overall gene repression state. These results are consistent with the H3K9ac expression pattern in the AD brain; it is unclear whether they are induced in AD due to gradually decreasing REST levels. REST is a master organizer of enzymes involved in histone acetylation and histone methylation 263, 265, and its role in the imbalance of histone methylation has not been clarified.

Since REST is lost at the MCI and AD stages, it is conceivable that the enzymatic complexes organized by REST will be disrupted under these conditions. However, in neurons where REST is absent, REST corepressor (CoREST) is expressed at high levels and exists in complexes with HDAC1, HDAC2, and LSD1 276, 277. The LSD1-CoREST complex mediates H3K4me1/2 demethylation and is commonly associated with silencing gene expression 277, 278. On the other hand, the CoREST complex is not recruited to its target loci without the REST scaffold 276, leading to an increase in H3K4me3 in the PFC of the AD brain due to a lack of demethylation catalyzed by LSD1 and its associated histone lysine demethylase 5A (KDM5A, an H3K4me3 demethylase) 279. Also, PFC neurons depend critically on lysine methyltransferase 2A (KMT2A, also known as MLL1) and mildly on lysine methyltransferase 2B (KMT2B, also known as MLL2) to maintain H3K4me3 levels at a subset of genes with an essential role in cognition and emotion, for example, at the ARC immediate early gene, which is an important mediator of synaptic plasticity and an AD susceptibility gene 280, 281.

These data indicate that improved activities of KMT2A and 2B may contribute to an increase in H3K4me3 in the PFC of the AD brain. By contrast, it is difficult to define how H3K4me3 is reduced in the hippocampus of the AD brain without REST. In the hippocampus, the LSD1-CoREST complex can most likely bind to the loci, recruit KDM5A, and cause the ensuing demethylation of H3K4me3 279, 282. Thus, when REST is lost, the recruitment of the LSD1-CoREST complex and KDM5A to REST-binding loci is region dependent 283. KMT2A/MLL1 and KMT2B/MLL2 have also been shown to mediate hippocampal H3K4 di- and trimethylation and are critical players in memory formation 284, 285. Therefore, it cannot be excluded that KMT2A and 2B become deficient due to DNA damage/rearrangement or fail to be recruited to target genes via ncRNA Mistral in the AD hippocampus and cause H3K4me3 reduction 286-288. Interestingly, LSD1 mediates the demethylation of histone H3K4me1/2 and H3K9me1/2, and its deficiency may lead to an increase in H3K9me2 in the AD PFC 289, 290. However, other studies revealed that in AD PFC, GLP/G9a (EHMT1/2), a REST-interacting protein that catalyzes H3K9me0 to H3K9me1 and H3K9me1 to H3K9me2, is significantly elevated 221. The H3K9me2 reduction in the hippocampus may be attributed to a region-dependent lack of GLP/G9a complex at the loci upon loss of REST; GLP/G9a complex is normally recruited to the loci by REST through chromodomain on Y-like (CDYL) and is required for memory consolidation 291-293. It is noteworthy that there exist other related histone methyltransferases and demethylases for H3K4 and/or H3K9 modifications 289, 294-296, but their functional roles are unclear and need further investigation.

Notably, the same set of genes within different brain regions may be enriched in different markers. For example, in PFC, H3K4me3 is enriched at the ARC gene locus 280, whereas in the hippocampus, H3K9me2 is enriched at this locus 269. The enrichment of H3K4me3 or H3K9me2 at the ARC locus in different brain regions reflects different activation or DNA repair states. However, H3K4me3 and H3K9me2 have also been reported to co-occur at the same locus, where PHF2 (an H3K9me2 demethylase) binds to H3K4me3 and recruits suppressor of variegation 39H1 (SUV39H1, an H3K9me2/3 methyltransferase) to coordinate H3K9me2/3 and H3K4me3 levels and thereby regulate their target gene expression 297. PHF2 promotes the expression of memory-related genes by epigenetically reinforcing the Tyrosine kinase receptor B (TRKB)-CREB signaling pathway 298. While the co-occurrence of H3K4me3 and H3K9me2 reflects the association of these two epigenetic markers, other factors can help determine whether the target gene is activated or inhibited. For example, H3K9ac, H3K4me3, and H3K9me2 concomitantly act on the same set of genes 299, suggesting that H3K9ac can disrupt the balance between H3K4me3 and H3K9me2 and cause activation of the target genes. Intriguingly, H3K9ac was enriched in cell death-promoting genes in AD, whereas H3K4me3 and H3K9me2 were enriched in cell protection and memory formation genes 236, 242. Thus, the overall outcome of simultaneous enrichment of all three markers (H3K9ac, H3K4ac, and H3K9me2) may have a deleterious effect on cells because the overactivation of a beneficial gene can be toxic. For example, it has been shown that inhibition of GLP/G9a (H3K9me2) rescues synaptic and cognitive functions by stimulating glutamate receptor expression 221; however, excessive glutamate receptor activity causes excitotoxicity and promotes cell death 300. This is consistent with previous findings that REST is involved in repressing the expression of glutamate receptors and ARC in addition to synaptophysin and PSD-95 to maintain synaptic homeostasis 301, and H3K4me3, H3K9me2, and H3K9ac are coordinated by REST 221, 236, 280.

#### 4.2.3. Cross-regulatory mechanism between histone methylation and acetylation

A chromatin-modifying complex containing PHF8 and TIP60 has been identified to be involved in the crosstalk between H3K9me2, H3K9acS10P, and H3K4me3 267, 269. Clinically, the mutation in PHF8 causes X-linked mental retardation (XLMR), while the mutation in TIP60 has been implicated in the pathogenesis of AD 269, 302. Mechanistically, at REST-bound promoters, PHF8 can bind to H3K4me3 (also RNA polymerase II) and demethylate H3K9me1/2 to H3K9me0 270, while TIP60 can bind to H3K9me3 and acetylate H3K9me0 to H3K9ac 266, 303. In addition, the PHF8 target gene SMCX (JARID1C/KDM5C) can bind to H3K9me3 and demethylate H3K4me3 to H3K4me1/2 265, 290. Furthermore, Wang et al. found that H3K4me3 facilitates the acetylation of both H3K9 and H4K16 304. H3K4me3 and H4K16ac were shown to be specifically recognized by the Bromodomain and PHD finger transcription factor (BPTF, also known as FAC1) and constitute a unique trans-histone modification pattern in the human genome 305. Besides, Katoh et al. found that FOXP3, an X-linked suppressor of autoimmune diseases, increases both H4K16ac and H3K4me3 at multiple FOXP3-activated genes by recruiting MOF (which interacts with MLL1) and displacing histone H3K4 demethylase PLU-1 (KDM5B or JARID1B) 306, 307. It has been reported that BPTF is a subunit of the nucleosome remodeling factor (NURF) complex, which mediates ATP-dependent chromatin remodeling 308. FOXP3 is a member of the forkhead transcription factor family, mainly expressed in a subset of CD4+ T cells and plays a suppressive role in the immune system 309. Interestingly, both BPTF and FOXP3 are associated with AD. BPTF protein was found in Hirano bodies and swollen dendrites in the hippocampus of AD patients 310, whereas FOXP3 contributed to chronic neuroinflammation and disease escalation in AD 311. Recently, Zhao et al. discovered that ectopic introduction of H3K27ac in the promoter region resulted in H3K4me3 enrichment around transcription start site (TSS) via Bromodomain-containing protein 2 (BRD2), while the presence of H3K4me3 at the promoter could not induce H3K27ac increase and failed to activate gene expression 312. However, deposition of H3K4me3 via KMT2B/MLL2 could remove the repressive marker H3K27me3 and DNA methylation 313.

In summary, H3K4me3 appears to play a coordinating role among the transcriptionally permissive and repressive markers. It interacts directly with several proteins, including PHF8, FOXP3, and BPTF, which further interact with TIP60, MOF, and other subunits of the NURF complex to form an H3K4me3-centered interaction network. Interestingly, while H3K4me3 is required for the transcription of DNA repair genes 314, 315, it prevents DNA repair at the damage sites 316. Thus, the coordination of PHF8, BPTF, and FOXP3 by H3K4me3 plays an important role in the early stage of AD, characterized by oxidative DNA damage 317. TIP60 is the core component of the DNA damage repair machinery 266, 270. In response to DNA damage, H3K9ac is downregulated in conjunction with the upregulation of H3K9me3 through KAP-1/HP1/SUV39H1. Subsequently, H3K9me3 activates TIP60, allowing the acetylation of H3K9 and ATM to prevent DNA damage 253, 303, 318. In contrast, under hypoxic conditions, PHF8 sustains the level of H3K4me3 and downregulates H3K9me1/2 through KDM3A to prevent DNA damage 270, 319-321. Interestingly, although PHF2 and G9a regulate the level of H3K9me2 in the opposite direction, both prevent DNA damage 322, 323, the discrepancy in these findings may be due to different cells used in the experiments 322, 323. Also, H3K9me2 has been shown to be enriched at CGIs and selectively inhibit the expression of the genes involved in BER 324, 325. Coincidently, H3K18ac and H3K27ac also play a role in BER in cooperation with uracil DNA glycosylase (UDG) and apurinic/apyrimidinic endonuclease 1 (APE1) 326, 327, and H3K27ac promotes oxidative stress-induced expression of noncoding RNA activated by DNA damage (NORAD), preserving genomic stability 328. Moreover, KDM4, which demethylates H3K9me3 at promoters denoted by H3K4me3 329, actively regulates the DNA damage response 330, 331. Finally, MeCP2, the intermediator of DNA methylation and histone modifications, can concomitantly downregulate H3K9ac and upregulate H3K9me3 levels and is involved in the DNA damage response 332, 333. Overall, histone acetylation (such as H3K9ac, H3K27ac and H3K18ac, and H4K16ac) and histone methylation (such as H3K4me3 and H3K9m2/3) are closely correlated with each other (Table 1) and form an internal regulatory network, which is involved in gene transcription and plays a role in DNA damage repair. Conversely, DNA damage and repair may disrupt the homeostatic state of the network. Therefore, maintaining homeostasis of the network and reorienting the expression profiles by interfering with these epigenetic markers may identify new AD drug candidates.

### 4.3. Noncoding RNAs (ncRNAs)

ncRNAs are a vast and diverse family of nonprotein-coding transcripts that are mainly divided into two groups: linear ncRNAs and circular ncRNAs. Linear ncRNAs can be further divided into two subgroups according to their lengths: small ncRNAs (sncRNAs, <200 nucleotides), including microRNAs (miRNAs, approximately 22 nucleotides), and long ncRNAs (lncRNAs, >200 nucleotides), including multiple natural antisense transcripts (NATs) 334, 335. Approximately 80% of the human genome is transcribed as noncoding transcripts, whereas less than 2% encodes proteins 336, 337. In the CNS, ncRNAs are particularly abundant. Approximately 70% of miRNAs and 40% of lncRNA genes are expressed in the brain 337. Remarkably, these noncoding transcripts are predominantly located in the nucleus, suggesting that their major function is epigenetic regulation 336, 338, 339.

A large number of ncRNAs are expressed in the CNS with precise temporal and spatial patterns 340-342, and a complex mesh of multitasking ncRNAs (especially miRNAs and lncRNAs) is deregulated and associated with the core pathological phenotypes of AD 343-348. Although the neurobiology of ncRNAs in the context of AD pathophysiology has been extensively reviewed 346, 347, 349, the underlying mechanisms remain incompletely understood due to their wide variety and complex nature. REST-related ncRNAs have recently been shown to play a key role in AD pathogenesis. REST is an epigenetic master regulator and a universal feature of normal aging in human cortical and hippocampal neurons, and its level is closely correlated with AD and longevity 236, 350. Also, REST and ncRNAs cross-regulate each other 351, 352. In this section, we review the current knowledge on the roles of REST-related ncRNAs in AD pathogenesis.

As mentioned above, REST binds to thousands of sites in the human genome and regulates a large array of coding and noncoding neuron-specific genes 262, 263. It forms complexes with histone-modifying enzymes and other chromatin remodeling partners 353-356. Especially, the REST complex can form a large complex with HOX antisense intergenic RNA (HOTAIR) and PRC2 351. HOTAIR is one of the most extensively studied lncRNAs in human cancer and various other diseases 357, 358. It facilitates protein-protein interactions, thereby regulating many pathophysiological processes, including epigenetic reprogramming, protein degradation, miRNA sponging, NF-κB activation, inflammation, immune signaling, and DNA damage response 357-360. HOTAIR also promotes the expression of DNA damage repair factors, including KU70, KU80, DNA-PKs, and ATM, by recruiting EZH2 to the target gene promoters and activates NF-κB by decreasing IκBα 361, 362. PRC2 is present in almost all eukaryotic cells and is involved in the epigenetic regulation of more than 2000 genes on 10 chromosomes; it is also responsible for H3K27me3 modification of 5%-10% histones 363. H3K27me3 and H3K4me3 often co-occur on bivalent promoters (poised HCPs) to keep the controlled genes expressed at basal levels. REST binding to the RE1 element increases the local level of H3K27me3 via PRC2 263 and decreases the H3K4me3 level via SMCX 265, 290 for gene silencing. Interestingly, the loss of function of REST can induce H3K27ac 364. Moreover, with aging, the level of H3K27me3 decreases while that of H3K4me3 increases, resulting in a progressive increase in gene expression 365, 366. Since H3K27me3 and H3K4me3 are involved in the DNA damage response and are associated with AD 169, 314, 367, it is conceivable that HOTAIR may play a role in the DNA damage response and AD pathogenesis by coordinating the levels of H3K27ac, H3K27me3, H3K4me3 and other epigenetic markers on the bivalent promoters by recruiting REST and PRC2 351.

Notably, REST is lost at the MCI and AD stages; therefore, it is unclear whether and how HOTAIR exerts its function at these AD stages 236, 368. At the prodromal AD stage, HOTAIR may cooperate with REST and PRC2 in the DNA damage response and gene silencing across the genome. For example, under ischemic conditions, which cause oxidative stress/DNA damage and are thought to be a prelude to AD 369-371, miR-132 (a neuron-protective microRNA downregulated in AD) expression is selectively silenced in hippocampal CA1 neurons by REST binding to its promoter rich in H3K9ac and H3K4me2 (another bivalent promoter) 352, 372. Interestingly, H3K27me3 is also rich in the promoter region of miR-132 373. In addition, polycomb group proteins (e.g., PRC2) are involved in ischemic tolerance 374. Considering these findings, it is possible that HOTAIR represses the transcription of neuron-protective target genes at the prodromal stage of AD by organizing PRC2 and REST, facilitating AD onset. This regulatory mechanism at the prodromal stage of AD can be tissue (region)-specific, probably due to variation in oxidative stress. For example, REST is involved in the downregulation of BDNF 375. However, forebrain ischemia induces downregulation of BDNF only selectively in hippocampal CA1 (like miR-132), but in CA3 and DG, the BDNF protein and other transcript versions are even upregulated 246. Also, histone acetylation patterns of H3K27ac, H3K9ac, and H3K14ac on BDNF promoters are different in CA1 compared with CA3 and DG, including decreased H3K27ac, increased H3K9ac and H3K14ac in CA1, increased H3K9ac, H3K14a, and H3K27ac in CA3, and no significant change of H3K9ac, H3K14ac, and H3K27ac in DG 246.

**Figure 2:**
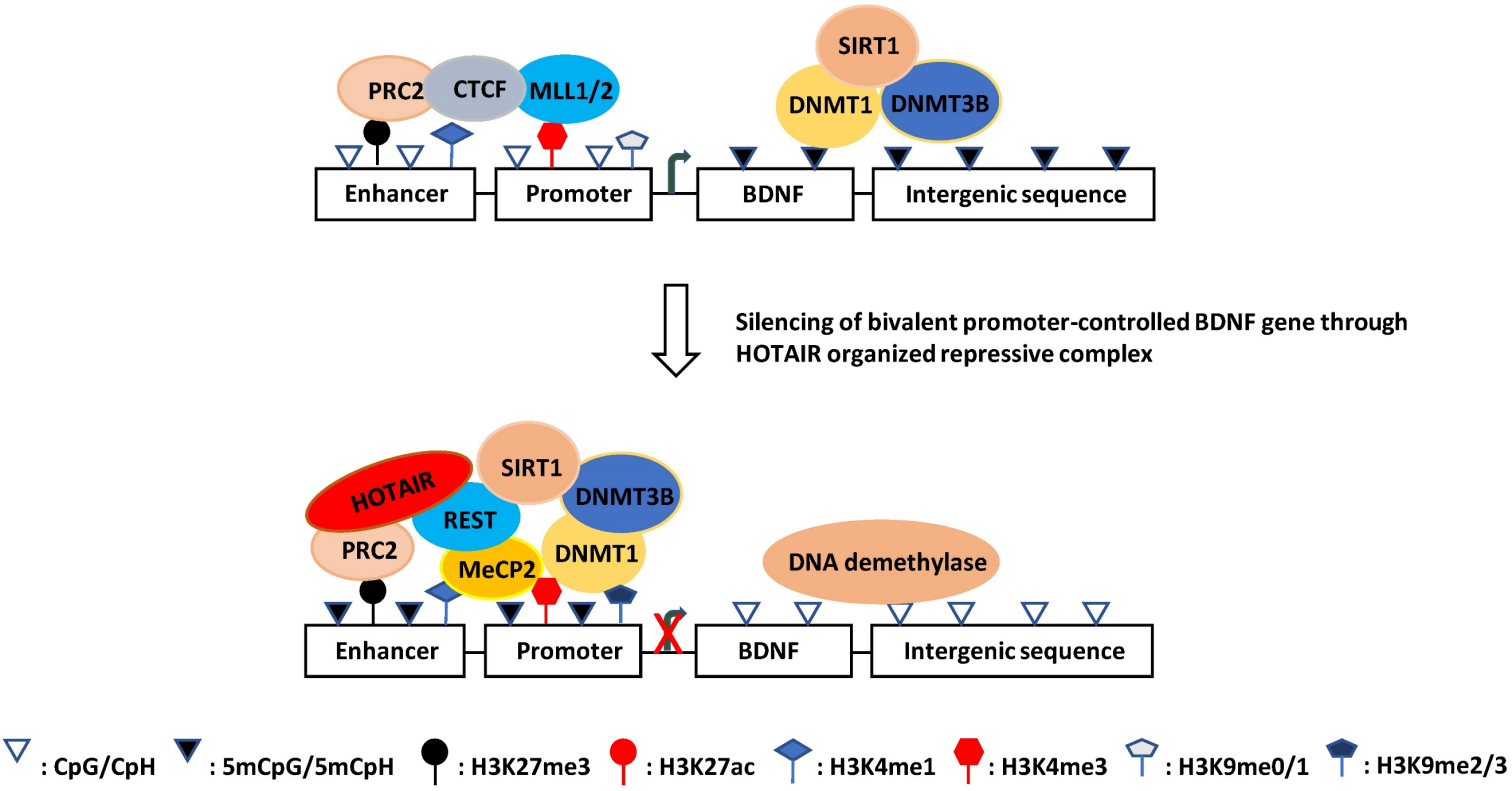
** Schematic illustration of the hypothesized mechanism of aberrant BDNF expression in AD.** DNA methylation at the damage sites recruits MeCP2 and HOTAIR-PRC2-REST complex which prevents coupling of promoter and enhancer resulting in transcription silencing. This event may be involved in the onset of AD.

Based on these observations, it appears that REST selectively represses its target genes in the hippocampus at the prodromal stage of AD by conjugating with HOTAIR in different hippocampal regions, where HOTAIR coordinates enzymatic complexes for specific chromatin remodeling. In keeping with this notion, many HOTAIR-organized molecules, including the REST-associated MeCP2-Sin3A-HDAC1 complex and PRC2-associated CBP-JMJD3 complex, have been observed at the BDNF promoter (Fig. 2) 181, 376-379. Moreover, REST and PRC2 have been shown to be involved in the downregulation of BDNF 375, 380, 381.

Finally, HOTAIR is one of the first described lncRNAs that can act in trans 382. Recently, HOTAIR was found to act as a competing endogenous RNA (ceRNA) by sponging miR-331-3p in gastric cancer 383, 384. Although it is unclear whether in the CNS, HOTAIR plays a similar role as in gastric cancer, two reports revealed that miR-331-3p is associated with AD 385, 386. Moreover, it has been shown that HOTAIR negatively regulates the cyclin-dependent kinase 5 regulatory subunit 1 (*CDK5R1*) gene, which encodes p35, the main activator of CDK5. The active p35/CDK5 complex is involved in numerous aspects of brain development and function, and its deregulation is associated with AD onset and progression 387. Recently, it was reported that HOTAIR negatively regulates the miR-455-3p/Nod-like receptor protein 1 (NLRP1) axis 388, whereas miR-455-3p has been shown to play a protective role against Aβ-induced toxicities and enhance cell survival and lifespan extension 389, 390.

In silico analysis for its target prediction showed the binding capacity of miR-455-3p with several AD-associated key genes, such as APP, Nerve growth factor (NGF), Ubiquitin specific peptidase 25 (USP25), p53 and DNA damage regulated 1 (PDRG1), Small mothers against decapentaplegic member 4 (SMAD4), Ubiquilin 1 (UBQLN1), SMAD family member 2 (SMAD2), Tumor protein p73 (TP73), Vesicle associated membrane protein 2 (VAMP2), HSPB1 Associated Protein 1 (HSPBAP1), and Neurexin 1 (NRXN1) 391, 392. It is unclear whether the regulatory mechanisms of HOTAIR on CDKR5 and the miR-455-3p/NLRP1 axis are dependent on its role as a ceRNA or as a scaffold for REST and PRC2. However, miR-455-3p, a potential biomarker for MCI and AD, has been shown to be upregulated at the MCI and AD stages, suggesting that it is derepressed, probably due to the depletion of REST at these AD stages. Furthermore, PRC2, the other partner of HOTAIR, also decreases with age, potentially promoting LOAD development through the dysregulation of APP and PS1 393. PRC2 has also been found to bind promiscuous RNA to scan for target genes that have escaped repression 394, 395. These results suggest that HOTAIR plays an important role in AD pathogenesis by coordinating REST and PRC2 as a scaffold and sponging miR-331-3p as ceRNA. Thus, HOTAIR may be another drug target for AD therapy (Table 1).

## 5. Loss of proteostasis

Proteins are important components of organisms and participate in all physiological functions. Thus, protein homeostasis must be tightly controlled to maintain fundamental biological processes and survival. However, as an organism ages, the proteome, like the genome, is easily disrupted and damaged 57, 396. Genomic instability and loss of proteostasis are closely interrelated. DNA damage may reduce the expression of proteins 33, and altered protein expression can inversely affect the DNA structure 249. Although genomic instability and loss of proteostasis are primary aging hallmarks, aging and its associated diseases are considered a snowballing phenotype of accumulated damaged or toxic proteins 22, 397. In this context, at the early and advanced stages of AD, alteration of protein synthesis machinery, including nucleolar chaperones, ribosome proteins, and elongation factors, has been observed in the frontal cortex and hippocampus 249, 398. Moreover, proteins related to many other biological processes are also aberrantly downregulated due to genomic instability in the AD brain 399-401.

### 5.1. Transcription factors in proteostasis

The regulation of proteostasis mainly includes protein production and degradation. For protein production, transcription factors (TFs) retrieve genetic information from the DNA to RNA together with other factors. Efficient transcription requires the initiation complex and the TFs that bind to the promoter sequence upstream of the TSS 402. TFs are readers of epigenetic markers, which may regulate transcription by many incompletely understood mechanisms, including stabilization of the initiation complex, destabilization of the chromatin structure, and conformational change of the promoter domain 403, 404. The binding of approximately 60% of TFs to their target genes is influenced by 5mC 207. Distinct profile patterns of chromatin features are related to different TF binding events 405. Conversely, transcription can shape the genome-wide methylome and histone acetylome patterns 148, 406. Of particular interest, computational analysis of the importance of various DNA-intrinsic and chromatin-associated features of ENCODE data showed that only H3K27ac, H3K4me2, H3K4me3, and H3K9ac are more reliable predictors of TF occupancy 405, whereas position weight matrix (PWM)-scores are among the most important features only for two TFs, namely, REST and CCCTC-binding factor (CTCF) 407.

REST and CTCF are abundantly expressed in the brain and are critical for memory 408, 409. Additionally, their binding sites are broadly distributed in the genome 207, 410-412, and their distribution patterns are markedly similar to each other when compared to those of other TFs 413, 414. Furthermore, REST and CTCF are involved in DNA integrity maintenance 270, 415-419 and depletion of DNA methylation 148, 420. In particular, REST and CTCF are critical for maintaining proteostasis 421, 422, and their defects are associated with AD pathogenesis 236, 423, 424. In the following section, we review these two TFs and their mechanisms in AD pathogenesis.

### 5.2. REST

REST is a master transcriptional regulator that controls approximately 2000 target genes 425. It is usually upregulated with aging in human cortical and hippocampal neurons, protecting them from oxidative stress and Aβ toxicity 236, 426. However, in MCI and AD, REST is lost from the neuronal nucleus and appears in autophagosomes together with pathological misfolded proteins 236, suggesting that REST degradation is accelerated in AD. However, whether the production of REST protein is also problematic in AD has not been investigated.

#### 5.2.1. DNMT1 and β-catenin in REST expression

**Figure 3:**
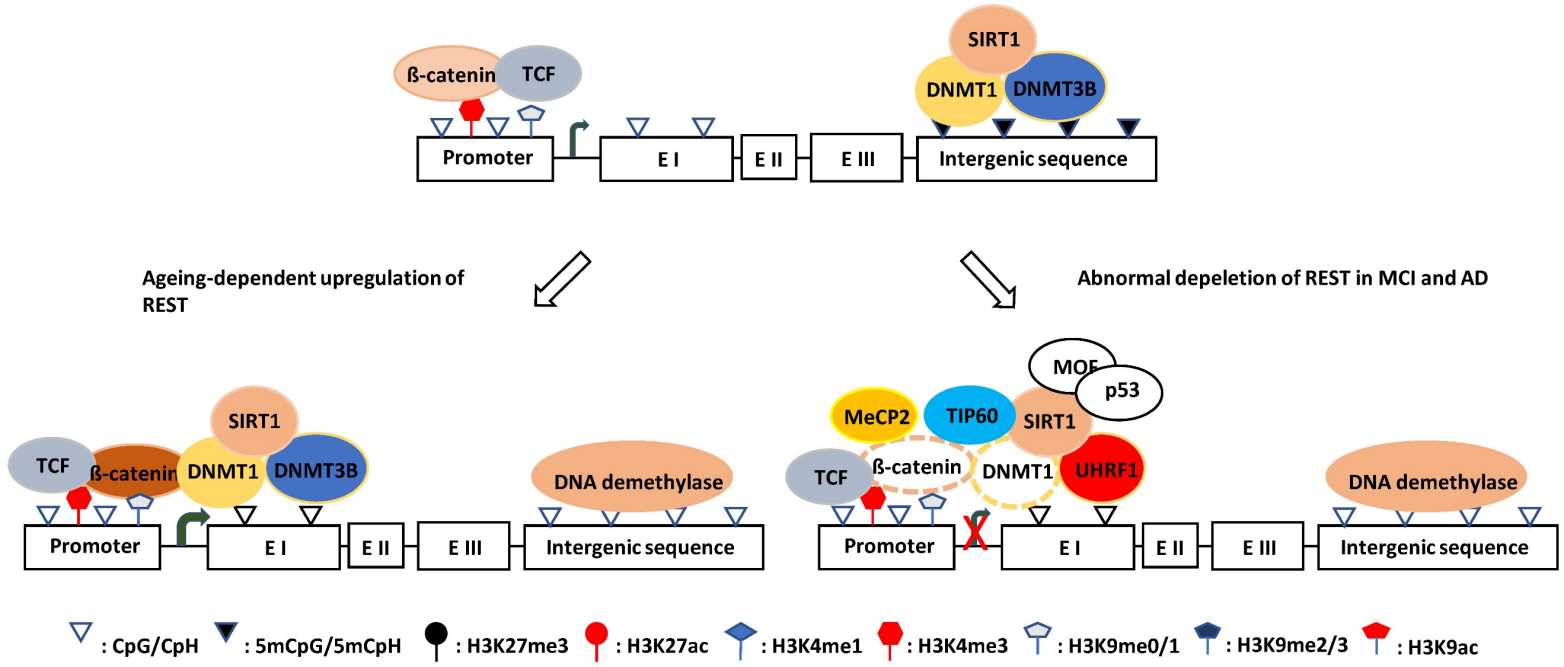
** Schematic illustration of the hypothesized mechanisms of REST expression in ageing and AD.** (left) Upregulation mechanism: DNMT1 shifts from non-CGI region to CGI regulatory element under oxidative stress and forms a ß-catenin stabilizing complex to prevent DNA methylation and increase ß-catenin/TCF activity thereby increasing REST transcription. (Right) Downregulation mechanism: DNMT1 is degraded when acetylated by TIP60 and ubiquitinated by UHRF1. which can lead to ß-catenin destabilization. Whereas, MeCP2 can reduce availability of ß-catenin for binding to DNMT1. Both cases will downregulated REST transcription. MOF and p53 prevent SIRT1 expression, which can aggravate DNMT1 degradation.

The canonical Wnt signaling pathway has been reported to regulate REST expression, and the β-catenin/TCF complex upregulates the REST gene transcription through a conserved element found in exon 1a 427. Tomasoni et al. found that REST regulates its own expression through the REST-TSC2-ß-catenin signaling pathway in neural cell models 428. Song et al. reported that DNMT1 and β-catenin mutually regulate each other, and DNMT1 upregulates β-catenin/TCF-driven transcription by forming a complex with β-catenin and LSD1, where LSD1 demethylates DNMT1 and stabilizes it 200, 429. Moreover, Funato et al. demonstrated that oxidative stress could activate β-catenin/TCF through the redox-sensitive association between thioredoxin and dishevelled 430, while O´Hagan et al. showed that, under chronic oxidative stress, DNMT1 (also DNMT3B and SIRT1) shifts from non-GC-rich genes and chromosome regions to CGIs 191. Oxidative stress can also promote DNMT1 enrichment at CGI promoters, which may then upregulate REST expression by forming a β-catenin stabilizing complex. This mechanism probably explains why the REST level increases with aging which is normally accompanied by chronic oxidative stress 431. Notably, DNMT1 may exert its function in nonenzymatic and enzymatic ways during this process. On the one hand, it can stabilize β-catenin as described, and on the other hand, it can catalyze DNA methylation on exon 1a and/or the promoter of the REST gene (Fig. 3). Consistent with this, a significant portion of tissue-specific differentially methylated regions (T-DMRs) are positively correlated with the expression of transcriptional repressor genes 432.

#### 5.2.2. UHRF1 in REST depletion

In contrast, the Wnt signaling pathway is defective 433, 434, and β-catenin is destabilized by phosphorylation in AD 435-437. However, the upstream signaling that induces phosphorylation-dependent degradation of β-catenin is unknown. There are reports showing that hypermethylated in cancer 1 (HIC1, a zinc finger transcriptional repressor) can attenuate the Wnt signaling pathway by recruiting TCF-4 and β-catenin to the nuclear bodies 438, while SIRT1 can deacetylate β-catenin and decrease its affinity for TCF4, thereby reducing its activity 439. It is unknown whether these two events initiate the degradation process of β-catenin. However, HIC1 is reduced with age by DNA methylation and H3K27me3 modification (trimethylated by PRC2 and demethylated by KDM6A/UTX and KDM6B/JMJD3) 440-442. Additionally, in elderly and senile AD patients, the expression of SIRT1 (also SIRT3 and SIRT6) in the hippocampus and saliva was 1.5-4.9-fold reduced compared with age-matched healthy individuals 443. Although DNA hypermethylation with increasing age can promote SIRT1 gene transcription through the downregulation of HIC1 440, SIRT1 mRNA was degraded through the ROS-Chk2-HuR pathway in elderly and senile AD patients 444. Therefore, it appears impossible that REST depletion is due to the direct action of HIC1 and SIRT1 on β-catenin, and its destabilization and degradation in AD need to be elucidated.

Oh et al. found that UHRF1 inhibits REST expression 445. UHRF1 is an E3 ligase that can bind to H3K9me3 and interact with DNMT1 at the promoter region of the REST gene 445. Most importantly, UHRF1 ubiquitinates DNMT1 and promotes its degradation when DNMT1 is acetylated by TIP60 202, 203. Considering the importance of DNMT1 for the stability and activity of β-catenin 200, it is conceivable that DNMT1 degradation may lead to β-catenin destabilization and inactivation, resulting in REST depletion. In line with this notion, the levels of SIRT1 in the hippocampus and parietal cortex of AD patients were reduced compared with controls 443, 446. Because TIP60 is negatively regulated by SIRT1 447-449, its reduction is expected to increase the TIP60 level, which can acetylate DNMT1 for degradation, which itself is a substrate of SIRT1 201. Thus, SIRT1 reduction combined with TIP60 upregulation aggravates DNMT1 acetylation and destabilization. However, this notion is challenged by the finding that in the early AD Drosophila brain, TIP60 is decreased while HDAC2 is increased before Aβ plaque formation 220; it is probably caused due to the feedback regulation of TIP60 by UHRF1. While TIP60 interferes with the USP7‑UHRF1 association and induces UHRF1 degradation in an auto‑ubiquitination‑dependent manner 450, UHRF1 recruits and ubiquitinates TIP60 for degradation to prevent its overaction 451, 452. Collectively, SIRT1 reduction may play a key role in REST depletion by destabilizing DNMT1 directly by deacetylating it or indirectly by stabilizing TIP60.

#### 5.2.3. MeCP2 in REST depletion

MeCP2 is widely believed to be a transcriptional repressor involved in methylation-associated gene inactivation 453, 454, although it can also positively regulate gene expression 455, 456 and plays an important role in CNS development, spontaneous neurotransmission, and short-term synaptic plasticity 454. Interestingly, MeCP2 is broadly expressed in mature human brain cells at all ages 457, binds to methylated cytosines (5mCpG and 5mCpA) with high affinity, and induces genome-wide histone deacetylation, especially on promoters, resulting in repression of many genes 457-459. For example, MeCP2 represses many long genes (>100 kb) with neuronal functions 207, 460. Abuhatzira et al. found that MeCP2 binds to the promoters of REST and CoREST genes despite their unmethylated state and is involved in the repression of their expression 380. However, whether MeCP2 contributes to REST depletion in MCI and AD has not been studied. It usually binds to methylated CGIs and represses gene expression mainly through a histone deacetylase complex composed of mammalian switch-independent 3A (mSin3A), HDAC1, and HDAC2 461, 462. The complex deacetylates acetylated histones, which are associated with a target gene, then changes the chromatin structure and leads to the repression of gene expression, showing a direct causal relationship between DNA methylation-dependent transcriptional silencing and the modification of chromatin 332, 461, 462. But at late AD stages, when REST is lost, HDAC1 and HDAC2 were decreased in the PFC and hippocampus of AD patients compared with controls 234, 235. Thus, it seems highly unlikely that REST depletion in MCI and AD is caused by MeCP2-mSin3A-HDAC1/2-mediated deacetylation of acetylated histones associated with the REST gene promoter.

Kimura and Shiota discovered that MeCP2 could form a complex with DNMT1 through the same transcription repressor domain, which mediates interaction with mSin3A-HDAC1/2 463. It was proposed that DNA methylation in vivo is maintained by the MeCP2-DNMT1 complexes 463. It is conceivable that the MeCP2-DNMT1 complex formation at the REST gene promoter interferes with the MeCP2-mSin3A-HDAC1/2 and the DNMT1-β-catenin complex formation and, therefore, may lead to MeCP2 acetylation and β-catenin destabilization/activity reduction, possibly inhibiting REST gene expression. It has been reported that SIRT1 reduction in AD increases acetylation of MeCP2, thereby preventing its binding to HDAC1 and SWI/SNF DNA helicase/ATPase (ATRX) and enabling its binding to DNMT1 458, 463-465. Interestingly, as competitors of DNMT1 binding, both MeCP2 and β-catenin regulate the expression of tau protein in an antagonistic manner to regulate the REST gene. While MeCP2 promotes tau expression and prevents REST expression, β-catenin does this in the opposite direction 466-468. Moreover, MeCP2 and β-catenin interact with p300/CBP and are regulated by the p300/CBP-SIRT1 switch 469, 470. Taken together, these observations suggest that MeCP2 contributes to REST gene silencing in MCI and AD by binding to DNMT1 and excluding β-catenin from the DNMT1-β-catenin complex and not by its interaction with the mSin3A-HDAC1/2 complex. This is consistent with a previous report that MeCP2 may mediate gene expression repression via a histone deacetylation-independent mechanism 471.

#### 5.2.4. Signaling integration in REST depletion

As described above, REST expression is controlled by β-catenin, UHRF1, and MeCP2. While β-catenin promotes REST expression, MeCP2 and UHRF1 inhibit its expression 380, 427, 445. Interestingly, all three molecules interact with DNMT1 200, 202, 203, 463. Given that the level of DNMT1 is positively correlated with the stability and activity of β-catenin, REST depletion in MCI and AD may be due to β-catenin deficiency induced by DNMT1 reduction or redistribution. In agreement with this notion, Wnt/β-catenin signaling is greatly suppressed in the AD brain, and its activation inhibits amyloid-β production and tau protein hyperphosphorylation in the brain 472. It has been proposed that a sustained loss of function of Wnt/β-catenin signaling underlies the onset and progression of AD 473.

DNMT1, together with DNMT3B and SIRT1, shifts from non-GC-rich genes and chromosome regions to CGIs 191, promoting local DNA methylation and can then be degraded or redistributed through UHRF1 or MeCP2 202, 203, 463. In response to DNA damage, molecules including Males absent on the first (MOF/MYST1/KAT8), TIP60, and p53 are also recruited to the damage sites 257, 266, 474-477. They are usually involved in DNA damage repair 257, 266, 475-477, but they may also regulate DNMT1. It has been shown that TIP60 accelerates DNMT1 degradation together with UHRF1 202, 203. In contrast, MOF and p53 have not been reported to be directly associated with DNMT1 degradation. However, MOF (and TIP60) can acetylate p53 at the DNA binding domain (K120), thereby inhibiting SIRT1 expression and promoting p300/CBP autoacetylation 478-480, which, in turn, can destabilize DNMT1 through TIP60 and MeCP2 447, 465. Besides, MOF is the major acetyltransferase for the expression of H4K16ac, which can recruit p53 via the MDC1-53BP1-BRCA1 complex to DNA damage foci 257, 481. Thus, the MOF-p53 axis may induce REST depletion by downregulating SIRT1, which is decreased in AD 443. Additionally, HDAC1, which can deacetylate p53 and thereby upregulate SIRT1, is decreased in AD 234, 235, 482. Of particular importance, MOF and p53 are also AD risk factors 483, 484.

Notably, MOF and p53 are substrates of SIRT1 448, 485-488, and p53 is also a substrate of HDAC1 482. SIRT1 is multifunctional and is an important player in the DNA damage response as a histone and non-histone deacetylase 489. For example, SIRT1 interacts with the MYST domain of MOF via its catalytic domain and deacetylates autoacetylated MOF, the major acetyltransferase for the expression of H4K16ac 257, 490, while SIRT1 also deacetylates H4K16ac 491. Although deacetylated MOF robustly binds to nucleosomes, becomes more active, and is more easily degraded, the acetylation of MOF decreases its binding ability and the global H4K16ac level 257, 448, 490. Interestingly, normal aging leads to H4K16ac enrichment, but AD entails dramatic losses of H4K16ac in the proximity of genes linked to aging and AD, which are especially enriched in the REST binding motif 248. Since SIRT1 is recruited to damaged CGIs together with DNMT1 and DNMT3B 191, 447, 448, it is possible that DNA damage-induced imbalance of the SIRT1/MOF ratio causes loss of H4K16ac at the gene regulatory elements, which may recruit REST and silence the gene. This mechanism may contribute to REST depletion because REST can regulate its own expression 428.

In summary, REST gene silencing is apparently related to the DNA damage response. An array of molecules, including DNMT1, TIP60, UHRF1, ß-catenin, MOF, p53, and SIRT1, play critical roles in the silencing process. These molecules may act in a cascade pathway or in an integrated platform and eventually cause β-catenin deficiency, resulting in REST depletion. Based on the above findings, REST depletion process appears to be as follows: (1) chronic oxidative stress causes DNA damage and DNA damage response, (2) continuous DNA damage and DNA damage response cause accumulation of DNMTs and DNA methylation at the regulatory elements, (3) MeCP2 binding to methylated CpGs and DNMT1 enhances DNA methylation and repels β-catenin from the β-catenin-DNMT1 complex, thereby inactivating β-catenin transcriptional activity, and (4) MeCP2-mediated gene silencing can be aggravated by TIP60/UHRF1-mediated DNMT1 degradation, MOF-mediated upregulation of p53 activity, and subsequent downregulation of SIRT1. Thus, MeCP2 plays a key role in REST depletion (Fig. 3). However, it is noteworthy that in addition to its multiple functions, MeCP2 also interacts with RNA binding fox-1 homolog (Rbfox) and the lncRNA Retinal non-coding RNA3 (RNCR3), affecting chromatin remodeling and mRNA splicing 492-494. Whether and to what extent the changes in mRNA splicing caused by MeCP2 are related to REST loss are unknown. However, an altered RNA spliceosome was reported to be associated with the expression of APP and TRKB 495, which are closely related to REST depletion 380.

### 5.3. CTCF

CTCF is a multifunctional protein in genome regulation and gene expression 411 that can bind to tens of thousands of genomic sites relying on distinct pathways compared with REST 496, 497. CTCF preferentially binds to unmethylated DNA sequences 496, 498, 499; it can bind to DNA damage sites and activate a cascade reaction resulting in DNMT1 inactivation and DNA demethylation 500. Moreover, CTCF interacts with histone modifiers, including HATs and HDACs, and is involved in histone modifications 501, 502. In this respect, CTCF is especially required to implement both H3K27ac and H3K27me3 503. Collectively, CTCF is a master organizer of chromatin structure, which plays a key role in DNA damage repair and gene transcription, and its defect may rewire genome-wide chromatin accessibility and have serious implications 504. Thus, the decline in CTCF with age may be an initial mechanism in AD pathogenesis 505, 506.

#### 5.3.1. APP and Tau

It has previously been reported that CTCF is required for the expression of APP and PAX6 423, 507, 508. PAX6, in turn, directly regulates the transcription of GSK-3β, which further catalyzes amyloid-β-mediated tau phosphorylation 509, 510. Since CTCF decreases with age, it does not seem to directly upregulate APP in AD 511, 512. However, the decline in CTCF might indirectly interfere with APP expression. For example, SMAD3 and SMAD4 have been shown to be specifically associated with the CTCF-APP promoter binding beta (APBbeta) complex to promote the TGF-beta-induced expression of APP 513. However, in another study, SMAD3 and 4 were shown to form nuclear complexes with SP1 in TGF-beta-mediated APP expression 514. Since the CTCF binding site overlaps with that of SP1 515, the decline in CTCF may enable the complex formation of SP1 with SMAD3 and 4 in regulating APP expression. Consistent with this notion, SP1 was upregulated in the cortex and hippocampus of the AD mouse brain, upregulating APP and tau 516, 517. Also, HDAC1, which interacts with SP1 and represses its activity, was reduced in the PFC and hippocampus of AD patients 234, 235, 518. REST, which interacts with and represses SP1, is also lost in MCI and AD 236, 519, 520.

#### 5.3.2. NPAS4

Neuronal PAS domain protein 4 (NPAS4) is a brain-restricted and activity-induced TF for synaptic excitation/inhibition (E/I) homeostasis 521. The expression of NPAS4 is synergistically repressed by REST and CTCF, with REST binding to the promoter and CTCF binding within intron I of the NPAS4 gene 521. Accordingly, the decrease in CTCF or loss of REST can increase the level of NPAS4 due to reduction or loss of gene repression. Interestingly, as mentioned earlier, the decline in CTCF and loss of REST may increase APP level, whereas APP can also upregulate the NPAS4 expression 522, 523. Thus, the decrease in CTCF and/or loss of REST may induce neuronal E/I imbalance by upregulating NPAS4 directly or indirectly via APP. Besides, APP can mediate tau phosphorylation, whereas APP-induced upregulation of NPAS4 can facilitate the autophagic clearance of tau 522. Thus, CTCF appears critical for the homeostatic maintenance of the APP-NPAS4-tau axis, and its decline or loss with age may cause APP/tau dyshomeostasis and NPAS4-mediated E/I imbalance, which usually occurs in the early stage of AD 524.

#### 5.3.3. PCDHs

Protoconherins (PCDHs) constitute the largest subgroup within the cadherin family of calcium-dependent adhesion molecules 525, 526. There are three large protocadherin clusters (PCDHα, PCDHß, and PCDHγ) that are highly expressed in the brain 525, 526 and are involved in CNS development and establishment of cell diversity and appropriate neural circuits 525-530. A decreased protocadherin expression was associated with severe dysregulation of dendritic morphology and arborization 531. Thus, PCDHα is critical for learning and memory 532, 533.

CTCF functions as an architectural protein that can mediate inter- and intra-chromosomal interactions, playing an important role in chromatin loop formation and 3D DNA topology regulation 534, 535. It is also the only well-defined mammalian insulator protein with properties to form long-range chromatin loops 536, which are often found between active promoters, enhancers, and CTCF binding sites 537. However, the exact function of CTCF at a given genomic site is unpredictable 496 and determined by the associated TFs and the location of the binding site relative to the TSS, enhancer, and promoter of the gene 496. Enhancers are the most salient noncoding DNA elements sprinkled throughout the genome that activate gene promoters and help orchestrate proper spatiotemporal gene expression 538, 539. In the brain, enhancers accurately regulate intricate expression programs across different neuronal classes, providing incredible cellular and functional diversity 540. Thus, enhancer dysregulation may induce different expression levels of their target genes and predispose or contribute to diseases 540.

Many studies have shown that CTCF is a master regulator of clustered PCDH genes and promotes their stochastic and combinatorial expression by mediating topological chromatin interactions between enhancers and promoters 531, 541-544. Recently, Tang et al. found that REST directionally forms base-specific interactions with neuron-restrictive silencer elements (NRSEs) within distal enhancers and target gene promoters, preventing CTCF-mediated long-range enhancer-promoter interactions and leading to the downregulation of clustered PCDHα proteins 545. Notably, CTCF binding to sites proximal to each promoter of PCDHα genes is demethylation dependent. Epigenetic methylation of CTCF binding sites (CBS) abrogates CTCF binding and abolishes its ability to bridge long-distance chromatin looping interactions between distal enhancers and their target promoters 546-548. It is, therefore, conceivable that the loss of REST causes an increase in PCDHα, although the gained methylation at CBS and a decrease in CTCF may antagonize this effect, likely contributing to AD pathogenesis. This notion was supported by the observation that PCDHγC5 was increased in neuronal hyperexcitation conditions upon Aβ treatment and in APP/PS1 transgenic mice 549.

#### 5.3.4. Arc and BDNF

In the brain, enhancers are necessary for genes that create cellular and functional diversity and activity-dependent gene expression associated with long-term potentiation (LTP) and homeostatic plasticity 540. Likewise, CTCF-mediated enhancer-promoter interactions are involved in the regulation of basal 550, 551 and activity-regulated genes (e.g., Arc, Npas4, Fos, Rgs2, and Nr4a2) 540. Interestingly, although these activity-regulated genes have variable physiological functions and have different enhancers controlling their transcription plasticity 540, their defects can similarly lead to AD 281, 523, 552-554.

The expression of BDNF and Arc is cell type- and stimulus-specific and is finely tuned 555-557. It has been shown that the expression of BDNF and Arc is regulated by CTCF 558, 559. However, Sams et al. found that CTCF is involved in the activity-dependent expression of BDNF and Arc but not under basal conditions 409. As mentioned above, BDNF basal expression is controlled by poised promoters of H3K27me3 and H3K4me3 181, whereas the basal expression of Arc is regulated by the PHF8-TIP60 complex, which specifically counteracts H3K9me2 to facilitate the formation of H3K9acS10P 267, 269. Thus, the decline in CTCF with age may cause a decrease in BDNF and Arc by downregulating the coupling between the poised promoters and enhancers. In support of this notion, BDNF was found to be decreased in the hippocampus, parietal cortex, and temporal cortex of AD patients 560-562. Additionally, Arc was reduced in the hippocampus of AD patients and most mouse AD models 563, 564 but was increased in some mouse AD models 565, 566. Since BDNF and Arc are vital for activity-dependent plasticity and memory consolidation 567-571, and the expression of approximately 100 genes associated with the pathophysiology of AD depends on Arc 281, it is plausible that the decline in CTCF may contribute to the development of AD by downregulating BDNF and Arc.

#### 5.3.5. MHC II

CTCF can act as a transcriptional activator or repressor and an insulator in a tissue-specific or conserved way 496. Genome-wide studies support CTCF as a global insulator 572. Insulators are classically defined by two experimental properties, enhancer blocking (EB) and barrier insulation, and CTCF is generally considered to function solely via the EB mechanism with no direct role in barrier insulation 414. For example, CTCF binds to the XL9 enhancer element at the major histocompatibility complex class II (MHC-II) locus between human leukocyte antigen-DRB1 (HLA-DRB1) and HLA-DQA1 genes (driven by divergent promoters) and blocks their expression 573-575. However, in many neuropathological disorders, including AD, the expression of MHC-II in microglia is markedly increased 576.

Microglia profoundly affect chronic inflammation in the brain and increase susceptibility to AD 577. Nikolic et al. found that upon toll-like receptor (TLR) stimulation, Ctcf-deficient macrophages produced normal levels of proinflammatory cytokines IL-12 and IL-6 but manifested a strongly impaired capacity to produce tumor necrosis factor (TNF) and IL-10 and the IL-10 family members IL-19, IL-20, and IL-24 578. Furthermore, human genetic data point to a key role of microglia in the development and progression of AD pathology. Impaired microglial activities and altered microglial responses to β-amyloid are associated with increased AD risk. Activated microglia can also be harmful to neurons, mediate synapse loss by engulfing synapses, exacerbate tau pathology, and secrete inflammatory factors 579. Thus, the decline in CTCF may contribute to AD pathogenesis by tuning macrophage function on MHC-II presentation and cytokine production.

#### 5.3.6. H19 and IGF2

Another example of the EB function of CTCF is that it can bind to the maternally imprinted control region (ICR) of the H19/IGF2 locus with a group of downstream enhancers, thereby blocking IGF2 expression while activating the expression of H19 580. In parallel, on the paternal allele, IGF2 expression is activated, while H19 expression is silenced by binding of MeCP2-mSin3A-HDACs to ICR in a methylation-dependent manner 581, 582. Furthermore, the decline in CTCF and/or biallelic methylation of ICR lead(s) to loss of imprinting (LOI) of IGF2 583, 584, while the biallelic hypomethylation of ICR causes LOI of H19 584. Interestingly, IGF2 and H19 are associated with aging and AD 583, 585-587. While a normal level of IGF2 correlates with memory performance, overexpression of IGF2 can induce cellular senescence 583, 588, which is linked to AD 589. In contrast, H19 promotes neuroinflammation by driving HDAC1-dependent M1 microglial polarization 590, 591.

Intriguingly, the LOI of IGF2 has also been reported in some regions of normal adult and whole fetal brains 592, suggesting that IGF2 is overexpressed in these brain regions. However, there is evidence showing that the IGF2 level is decreased in the hippocampus of AD patients and a mouse model of this disease, and ectopic expression of IGF2 in the hippocampus of transgenic aged mice reduces Aβ plaques and reverses memory deficits 593. Considering that LOAD is age-dependent, these findings may indicate that the LOI of IGF2 with age induces overexpression of IGF2, which, in turn, causes cellular senescence and subsequent IGF2 reduction. As such, the decline in CTCF may contribute to AD pathogenesis through the LOI of IGF2. In contrast, H19 expression was drastically reduced in the adult brain compared to the fetal brain and was detectable only in the pons and globus palludus 592. However, Zhang et al. found that H19 knockdown promotes viability and inhibits oxidative stress in Aβ_25-35_-expressing PC12 cells by regulating the expression of miR-129 and high-mobility group box 1 (HMGB1), suggesting that H19 may be overexpressed in the AD brain 594. H19 overexpression is caused by the biallelic hypomethylation of ICR in cancer 584. There are no reports on whether and how biallelic hypomethylation of ICR occurs in the AD brain. However, oxo8dG and 5hmC in CGIs can induce DNA hypomethylation by inhibiting DNA methylation or activating the DNA demethylation processes 193. Additionally, there is evidence showing that H19/IGF2 methylation states are potentially relevant to hippocampal structure and function across the life course 595, 596. Taken together, the decline in CTCF may cooperate with the gained methylation on ICR at the initial stage of AD to induce LOI of IGF2, which then may cause cascade events resulting in LOI of H19.

#### 5.3.7. Other proteins

CTCF mediates POLD1 (the catalytic subunit of DNA polymerase δ) transcription, and its decline with age has been shown to accelerate the progression of cell aging and cognitive impairment in AD 505, 597. Also, a reduction in CTCF upregulates p16INK4a, which is involved in premature senescence 598. CTCF shares DNA binding sites in post-mitotic cells with cohesin 496, a ring-shaped complex composed of four core subunits, Structural maintenance of chromosome 1 (SMC1), SMC3, RAD21, and stromal antigen 1 (STAG1), and several cohesin-associated proteins 599. Cohesin is associated with DNA replication and sister chromatid cohesion during the S, G2, and M phases of the cell cycle. Also, it has intrinsic roles in maintaining the neuronal post-mitotic state, which is often disrupted by cohesin impairment and causes the aneuploidogenic phenotype of AD 600. As with CTCF, cohesin decreases with age and is probably the reason for the aneuploidogenic phenotype of AD. CTCF co-occupies sites with Forkhead box A1 (FOXA1) and the estrogen receptor (ER) which are also associated with AD 601-603, suggesting that it facilitates their transcriptional activation 496. CTCF also binds promiscuous RNA for genome organization 604. These findings indicate that CTCF is a key TF that may regulate the expression of many AD-associated proteins by cooperating with diverse interaction partners (Table 2).

In summary, REST and CTCF are a pair of TFs closely associated with many biological processes. Although they bind to different consensus sequences and have no reported physical interactions, they are spatially distributed near each other in the genome and have fine-tuned cooperation in maintaining genomic integrity and gene expression. REST homeostasis maintenance is dependent on DNMT1, whereas the homeostasis of REST and CTCF is required for decreasing DNA methylation. In response to DNA damage, they may play a compensatory role in the damage repair by keeping DNMT1 and its activity at the homeostatic level. However, in response to neuronal activity stimuli, REST and CTCF may play antagonistic or synergistic roles depending on their binding location and methylation state. Therefore, when REST and CTCF are absent or present at low levels, physiological processes associated with them may not function. Especially, DNA damage can be aggravated, and the neuronal E/I balance can be disrupted, causing cell death, and thereby contributing to AD pathogenesis.

## 6. Discussion

AD is the most prevalent form of dementia and is associated with many risk factors. This review highlights the primary hallmarks of aging in AD pathogenesis. Among the four primary aging features, three are related to genomic DNA, in agreement with the fact that the genome is a dynamic information and communication system sensitive to external and internal genotoxic stresses 605, 606. In particular, ROS can accelerate genomic instability and telomere attrition 51, 80, 81, 607, thereby deregulating epigenetic modifications 191, 266, 270, 608 and protein expression profiles 609, 610.

AD is characterized by SNV 6, 16, which is positively correlated with oxidized DNA signatures 611, 612. The accumulation of oxyradical-associated DNA damage in the hippocampus, midbrain, and caudate putamen is greater than in other regions 46, 63, 64, especially the hippocampus is vulnerable to damage at the early stages of AD 613. Since reactive species are signaling molecules that reflect cellular activity, brain regions that are more active and produce high ROS levels are more easily damaged, although there are autonomous and nonautonomous antioxidants to protect them 61, 63, 70-78. In agreement with above observations, DNA damage-associated DNA methylation and aberrant histone modifications in the AD hippocampus have been reported 168, 169, 236-238, 275. DNA methylation and histone modifications are well-established biomarkers that reflect the biological age of any tissue and predict early disease risk and mortality 207, 614, 615.

Under chronic oxidative stress, HCPs with low expression (H3K27me3 and H3K4me3) become hypermethylated, while HCPs with high expression (H3K27ac and H4K20me1) remain unmethylated 191, 209. De Jager et al. reported that DNA methylation at the strong promoters that drive fundamental cellular processes of neurons and glia in the healthy brain is not effectively altered in AD. Rather, methylation changes appear to primarily affect genomic regions that are weakly transcribed or inactive in the healthy older brain 164. These observations are consistent with the finding that the most prominent alteration of DNA methylation in AD occurs mainly at hypomethylated or unmethylated promoters or enhancers 164, while the global variation of DNA methylation is not well defined 194, 616-619. Interestingly, these hypomethylated or unmethylated promoters or enhancers are usually occupied by REST and CTCF in healthy cells 207, 496, 498, 620-623, which have been shown to protect against DNA methylation 148, 420, 624. While CTCF protects against DNA methylation through a cascade reaction resulting in DNMT1 inactivation, it is unclear how REST performs this function. It likely prevents DNA methylation by recruiting KMT2B/MLL2, which catalyzes H3K4me3 187, 313, 625. Therefore, the reported aberrant DNA methylation in AD may be induced by the depletion of REST and a decrease in CTCF.

Although H3K4me3 prevents DNA methylation, it is widely accepted that DNA methylation can direct histone acetylation, especially at sites occupied by REST and PRC2 332, 626, 627. Interestingly, the reported histone acetylation markers in AD are associated with the DNA repair response and are most likely induced by DNA methylation. MeCP2 may play a critical role as it can bind to the mSin3A-HDAC1/2 complex via methylated CpG, stimulating genome-wide histone deacetylation 458, 459, 461, 462 and also regulates DNA methylation by binding to DNMT1 in a mutually exclusive manner 463. However, in response to DNA damage, HATs, such as MOF and TIP60, can be recruited to the damaged sites by molecules other than MeCP2 and then interact with H3K9me3 266, 628-630. MOF specifically catalyzes H4K16ac 257, 631, whereas TIP60 can catalyze H3K9ac, H3K14ac, and H2AK5ac 238, 266, 475, 476. Thus, DNA methylation-directed histone deacetylation and MOF-TIP60-mediated histone acetylation play antagonistic roles at the damaged sites. MeCP2 downregulates H3K9ac in the DNA damage response via HDAC1/2 332, 333; however, when MeCP2 or HDAC1/2 is decreased, the H3K9ac level is expected to increase. Indeed, MeCP2 was reduced in the AD hippocampus 632, and HDAC1 and HDAC2 were generally downregulated in the AD brain 234, 235. This explains H3K9ac upregulation in the AD PFC and suggests that H3K9ac in the AD hippocampus may increase depending on MeCP2 and TIP60 levels.

H3K27ac is upregulated in parallel with H3K9ac in the AD brain. H3K27ac is catalyzed by p300/CBP and deacetylated by HDAC1/2 229, 241, 633, whereas MeCP2 can recruit p300/CBP and HDAC1/2 (with different domains) to methylated CpGs 464, 634. It is conceivable that the elevation of H3K27ac in AD is caused by loss of HDAC1/2 and local enrichment of p300/CBP. Given that MeCP2 binds to DNMT1 and regulates DNA methylation 463, the level of H3K27ac is correlated with DNA methylation. The finding supports the contention that p300/CBP and PRC2 are bound at unmethylated CGI loci occupied by REST and CTCF 178, 179, 184, 208, 363, 635, 636, which prevent DNA methylation and interact with PRC2 637, 638. CTCF was reported to be required for PRC2 stabilization and H3K27me3 expression 503, which in turn is needed for repressing HOTAIR production 639. HOTAIR forms a scaffold with REST and PRC2 in a gene-repressive complex 351, 640. Interestingly, HOTAIR also interacts with DNMT1 641. Therefore, CTCF and REST may play a critical role in DNA methylation-directed histone acetylation by cooperating with MeCP2, PRC2, p300/CBP, TIP60, and MOF.

It has been reported that DNA methylation can exclude H3K4me3, which is catalyzed by CXXC proteins, such as KMT2A/MLL1 and KMT2B/MLL2 642-644. On the contrary, DNA methylation can upregulate H3K9me2/3 concomitant with the downregulation of H3K9ac through the MeCP2-SUV39H1 complex 332, 645. Although G9a also catalyzes H3K9me2, MeCP2 was reported to repress its activity 221, 646. As previously mentioned, DNA methylation is increased in the PFC and hippocampus of AD patients, whereas H3K4me3 and H3K9me2 are significantly elevated in the PFC 221, 271, 273, 274 but downregulated in the hippocampus and entorhinal cortex 169, 249, 275. MeCP2 is positively correlated with H3K9me2 and robustly expressed in the PFC but is downregulated in the hippocampus of AD patients 632, 647. Therefore, it is understandable that H3K9me2 is increased in the PFC but is decreased in the hippocampus of AD. However, the reason for the H3K4me3 increase in the AD PFC is not known, it was expected to decrease as in the AD hippocampus due to aberrant DNA methylation. Notably, the H3K4me3 level is closely related to H3K9me2/3 through the PHF2-SUV39H1 complex 297. MeCP2 can also interact with chromodomain helicase DNA binding protein 1 (CHD1) and poly (ADP-ribose) polymerase 1 (PARP1), which bind to H3K4me3 648, 649. These data suggest that the PHF2-SUV39H1 complex and MeCP2 can prevent H3K4me3 exclusion by DNA methylation and, therefore, create a bivalent intermediate state, especially under DNA damage conditions. Furthermore, H3K9me3 can inversely upregulate DNMT1-mediated DNA methylation 650, while H3K4me3 inhibits *de novo* DNA methylation 187, 651. Thus, although DNA methylation may play a leading role in the regulation of histone methylation, there is a feedback loop and the crosstalk with H3K4me3, likely resulting in many intermediate states of these epigenetic markers. This notion is supported by the presence of many histone methylation markers, including H3K4me2, H3K9me2, H3K27me3, H3K36me2, H3K36me3, H3K79me2, H4K20me2, H4K20me3, and their related HMTs and HDMs at DNA damage sites 652.

In summary, age-dependent DNA damage, telomere attrition, and DNA repair processes can progressively change the overall pattern of DNA methylation and histone modifications 653-656. A dynamic network that coordinates DNA methylation and histone modifications may form at CGI regulatory elements, which are more susceptible to oxidative stress. This network contains HOTAIR, REST, and PRC2 as core components, with HOTAIR scaffolding REST and PRC2 361, 657, 658, REST scaffolding CoREST and MeCP2, and CoREST and MeCP2 scaffolding more epigenetic enzymes that interact with CTCF. Among them, only HOTAIR expression is not changed with age and sex 641, whereas CTCF, PRC2, and MeCP2 are decreased with age 393, 505, 632, and REST is increased with age but decreased in MCI and AD 236. CoREST and its associated LSD1 and HDAC1/2 decrease with differentiation 659, but in neurons where REST is absent, CoREST is expressed at high levels 276, 277. In addition, PRC1, which catalyzes the monoubiquitination of histone H2AK119 (H2AK119ub1) 393, 660, 661, was reduced and associated with AD 660, 661. PRC1 may be another core component of the network due to its interaction with PRC2 and REST 637, 662.

Most of the components of the network exhibit age-related decrease in parallel with the accumulation of DNA damage and are closely associated with the overall upregulation of DNA methylation and dysregulation of histone modifications at the gene regulatory elements. Given that DNA methylation plays a dominant role in rebuilding the landscape of histone modifications and in REST depletion, it is tempting to propose that DNMTs, MeCP2, and SIRT1 may be potential drug candidates for AD therapy. HDAC inhibitors (HDACis), including that of SIRT1, have been tried as candidate drugs for AD therapy. Although HDACis have shown promising results in mouse AD models, their nonspecific nature could interfere with REST-mediated neuroprotective pathways that require histone deacetylation 236, 663. This is consistent with the fact that SIRT1 plays a key role in the expression of REST and other proteins essential for the DNA damage response and cell survival. Because of the importance of SIRT1 in AD pathogenesis and its decrease with age, in the future, pharmacotherapy directed toward histone modifications could be employed to target the network of genes regulated by SIRT1 reduction 218, 277, 664.

**Figure 4:**
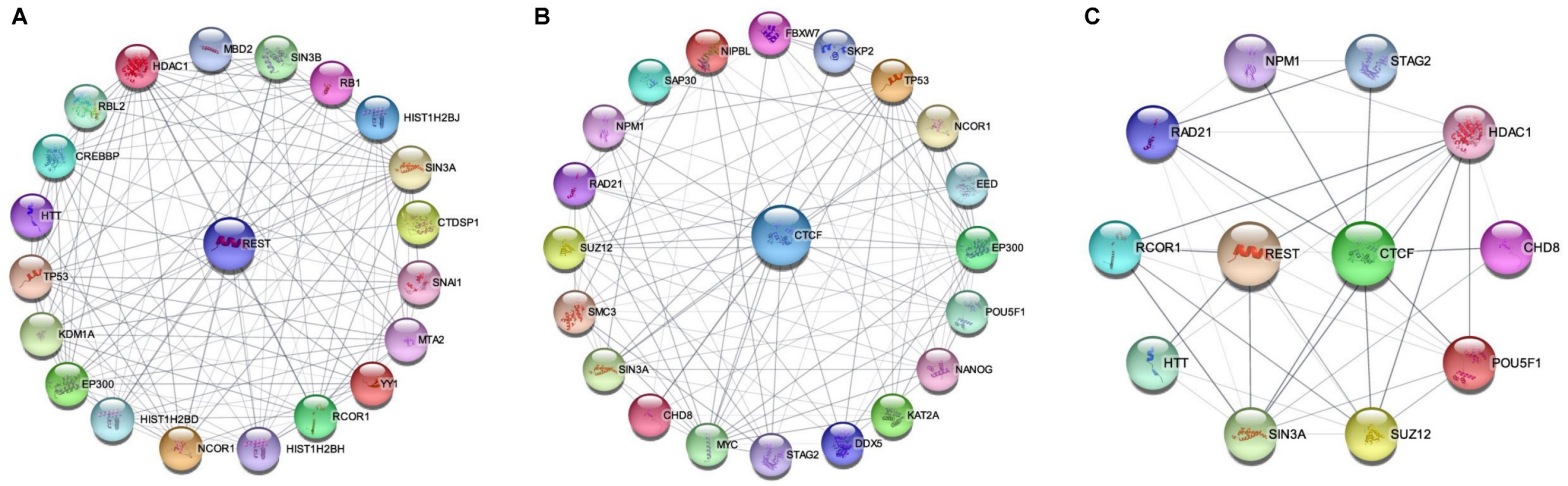
** STRING network of REST and CTCF interaction proteins and REST-CTCF coupling through their interaction partners drawn using Cytoscape (v3.7.1). (A)** REST Interaction partners; **(B)** CTCF interaction partners; **(C)** REST-CTCF coupling through their interaction partners. CHD8: Chromodomain Helicase DNA Binding Protein 8; CREBBP: CREB Binding Protein; CTDSP1: Carboxy-terminal Domain RNA Polymerase II Polypeptide A Small Phosphatase 1; DDX5: DEAD-Box Helicase 5; EP300: E1A Binding Protein P300; EED: Embyonic Ectoderm Development; FBXW7: F-Box And WD Repeat Domain Containing 7; HDAC2: Histone Deacetylase 2; HIST1H2BD: Histone Cluster 1 H2BD; HIST1H2BH: Histone Cluster 1 H2BH; HIST1H2BJ: HistoneCluster1 H2BJ; HTT: Huntingtin; KAT2A: Lysine Acetyltransferase 2A; KDM1A: Lysine Demethylase 1A: MBD2: Methyl-CpG Binding Domain Protein 2; MTA2: Metastasis Associated 1 Family member2; MYC: Myc Proto-Oncogene, BHLH Transcription factor; NANOG: Nanog Homeobox; NCOR1: Nuclear Receptor Corepressor 1; NIPBL: NIPBL Cohesion Loading factor; NPM1: Nucleophosmin 1; POU5F1: POU Class 5 Homeobox 1; RAD21: RAD21 Cohesion Complex Component; RB1: RB Transcriptional Corepressor 1; RBL2: RB Transcriptional Corepressor Like 2; RCOR1: REST Corepressor 1 (CoREST); SAP30: SIN3A Associated Protein 30; SIN3A: SIN3 Transcrition Regulatory Family Member A; SIN3B: SIN3 Transcrition Regulatory Family Member B; SKP2: S-Phase Kinase Associated Protein 2; SMC3: Structural Maintenance Of Chromosomes 3; SNAI1: Snail Family Transcriptional Repressor 1; STAG2: Stromal Antigen 2; SUZ12: SUZ12 Polycomb Repressive Complex 2 Subunit; TP53: Tumor Protein P53; YY1: YY1 Transcription Factor.

**Figure 5:**
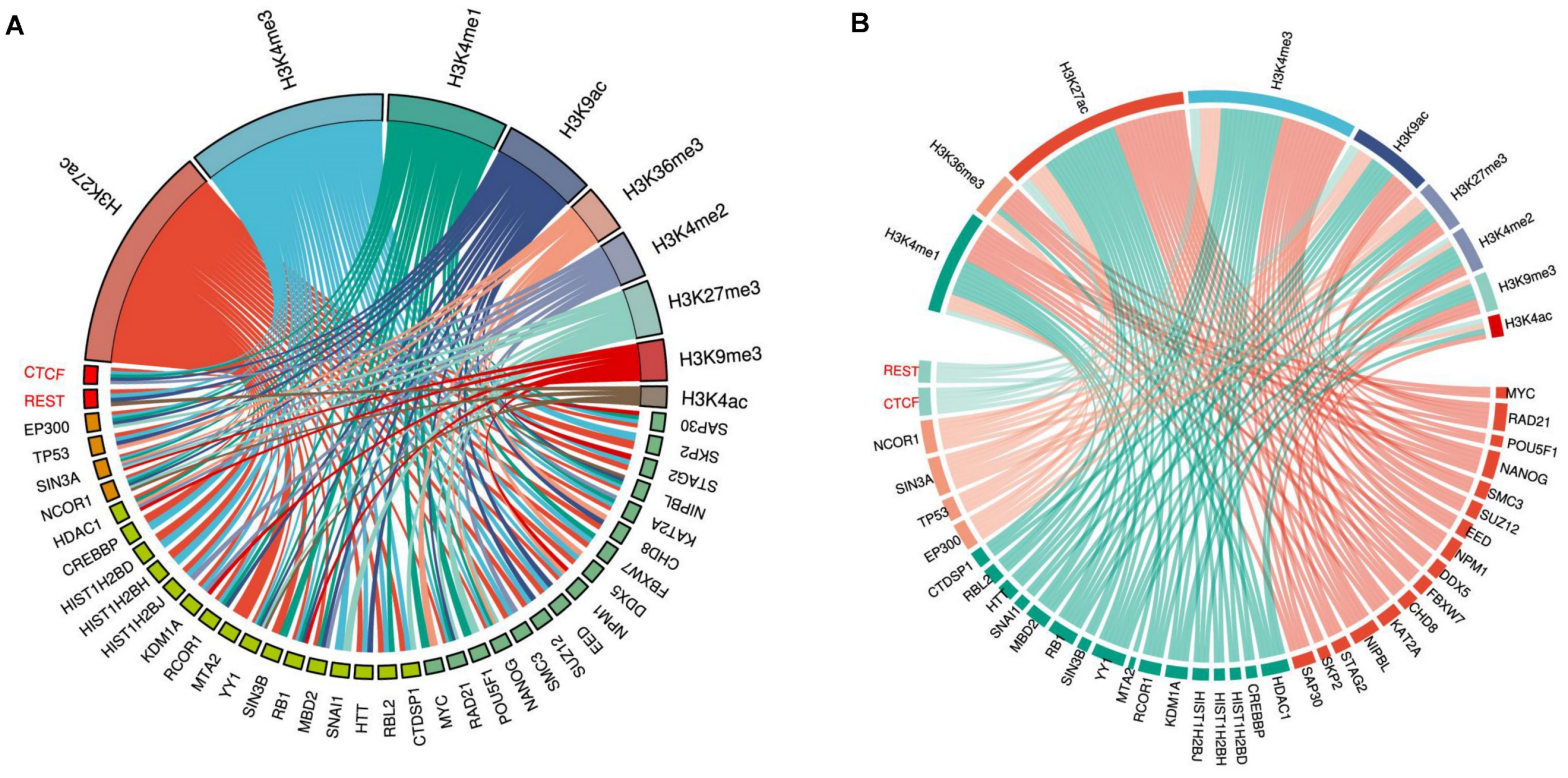
** Schematic illustration of correlation between REST, CTCF and their interaction proteins and selected epigenetic markers. (A)** Epigenetic markers of the genes related to neural and stem cells are recruited from http://dbtoolkit.cistrome.org, and the mode diagram was drawn using Goplot R package; **(B)** The mode diagram was drawn using Circlize R package.CHD8: Chromodomain Helicase DNA Binding Protein 8; CREBBP: CREB Binding Protein; CTDSP1: Carboxy-terminal Domain RNA Polymerase II Polypeptide A Small Phosphatase 1; DDX5: DEAD-Box Helicase 5; EP300: E1A Binding Protein P300; EED: Embyonic Ectoderm Development; FBXW7: F-Box And WD Repeat Domain Containing 7; HDAC2: Histone Deacetylase 2; HIST1H2BD: Histone Cluster 1 H2BD; HIST1H2BH: Histone Cluster 1 H2BH; HIST1H2BJ: HistoneCluster1 H2BJ; HTT: Huntingtin; KAT2A: Lysine Acetyltransferase 2A; KDM1A: Lysine Demethylase 1A: MBD2: Methyl-CpG Binding Domain Protein 2; MTA2: Metastasis Associated 1 Family member2; MYC: Myc Proto-Oncogene, BHLH Transcription factor; NANOG: Nanog Homeobox; NCOR1: Nuclear Receptor Corepressor 1; NIPBL: NIPBL Cohesion Loading factor; NPM1: Nucleophosmin 1; POU5F1: POU Class 5 Homeobox 1; RAD21: RAD21 Cohesion Complex Component; RB1: RB Transcriptional Corepressor 1; RBL2: RB Transcriptional Corepressor Like 2; RCOR1: REST Corepressor 1 (CoREST); SAP30: SIN3A Associated Protein 30; SIN3A: SIN3 Transcrition Regulatory Family Member A; SIN3B: SIN3 Transcrition Regulatory Family Member B; SKP2: S-Phase Kinase Associated Protein 2; SMC3: Structural Maintenance Of Chromosomes 3; SNAI1: Snail Family Transcriptional Repressor 1; STAG2: Stromal Antigen 2; SUZ12: SUZ12 Polycomb Repressive Complex 2 Subunit; TP53: Tumor Protein P53; YY1: YY1 Transcription Factor.

Another approach would be to prevent the downregulation of REST, CTCF, and other related core components of the network, which play a key role in maintaining homeostasis of DNA methylation and histone modifications. To this end, we have further analyzed the related molecules interacting with REST and CTCF by bioinformatics methods. Figure 4 highlights the molecules which mediate the coupling of REST and CTCF. We found that the expression of REST, CTCF, and their interaction partners is positively correlated with H3K27ac and H3K4me3 (Fig. 5; Table S1). Thus, maintenance of the homeostasis of the two epigenetic markers, H3K27ac and H3K4me3, may play a key role in maintaining REST and CTCF levels, and therefore represent promising drug target candidates.

## Supplementary Material

Supplementary table.

## Figures and Tables

**Table 1:**
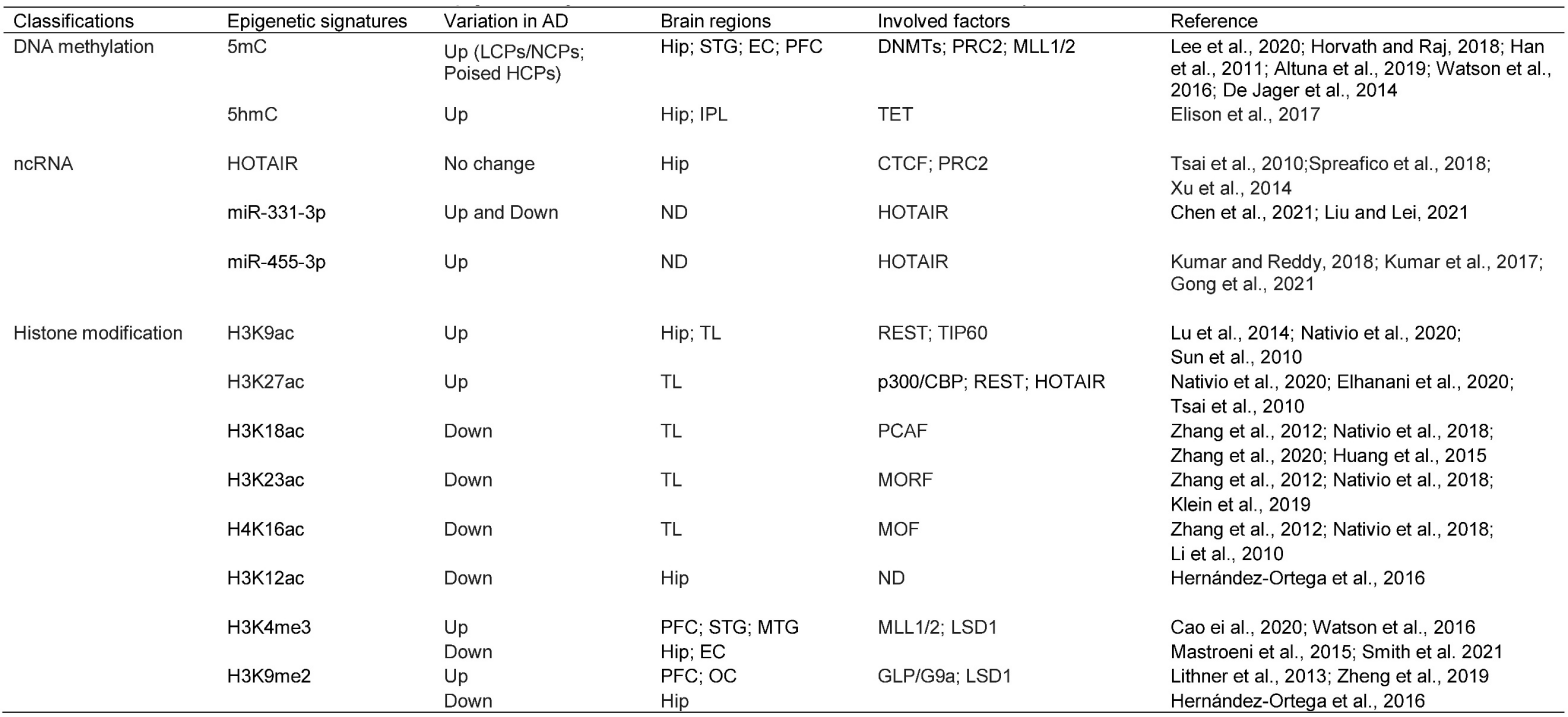
Overview of the epigenetic signatures in AD, their distribution and related enzymes or factors

**Abbreviations:** 5mC: 5-Methylcytosine; 5hmC: 5-Hydroxymethylcytosine; HOTAIR: HOX antisense intergenic RNA; H3K9ac: Histone H3 lysine 9 acetylation; H3K27ac: Histone H3 lysine 27 acetylation; H3K18ac: Histone H3 lysine 18 acetylation; H3K23ac: Histone H3 lysine 23 acetylation; H4K16ac: Histone H4 lysine 16 acetylation; H3K12ac: Histone H3 lysine 12 acetylation; H3K4me3: Histone H3 lysine 4 trimethylation; H3K9me2: Histone H3 lysine 9 dimethylation; LCPs: low-intensity CpG promoters; NCPs: non-CGI promoters; HCPs: High-intensity CpG promoters; Hip: Hippocampus; STG: Superior temporal gyrus; EC: Entorhinal cortex; PFC: Prefrontal cortex; IPL: inferior parietal lobe; TL: temporal lobe; MTG: Medial temporal gyrus; OC: Occipital cortex; DNMT: DNA methyltransferase enzyme; PRC2: Polycomb repressive complex 2; MLL1/2: Mixed lineage leukemia1/2; TET: Ten-eleven translocation; CTCF: CCCTC-binding factor; REST: Repressor element 1 (RE1)-silencing transcription; TIP60: Tat-interacting protein of 60 kDa; p300/CBP: p300/CREB binding protein; PCAF: p300/CBP-associated factor; MORF: MOZ-related Factor; MOF: Males absent on the first; ND: Not determined; LSD1: Lysine-specific demethylase 1.

**Table 2:**
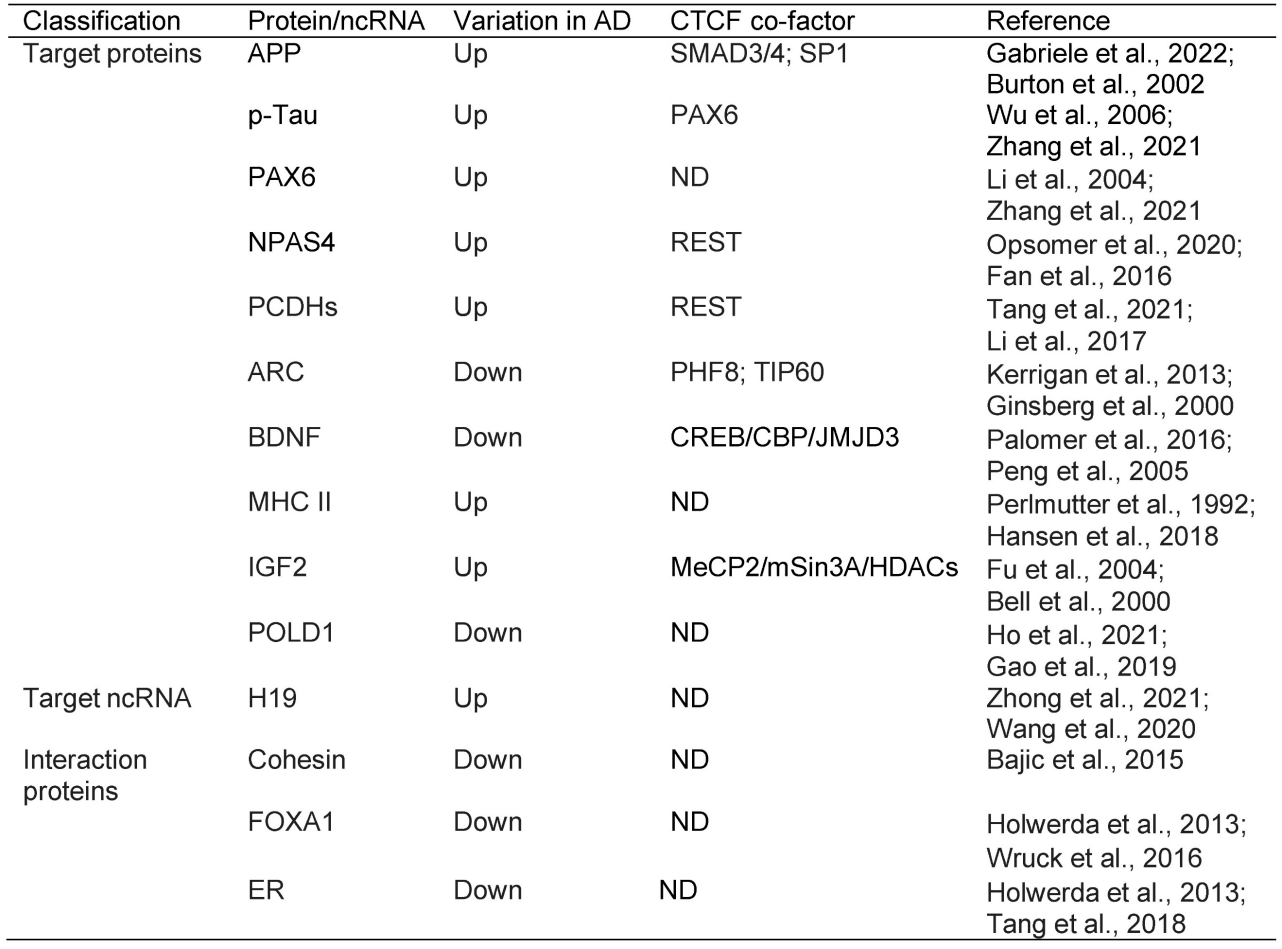
Overview of the AD-associated proteins/ncRNA regulated by CTCF and its co-factor

**Abbreviations:** APP: Amyloid precursor protein; p-Tau: Phosphorylated Tau; PAX6: Paired box 6; NPAS4: Neuronal PAS domain-containing protein 4; PCDHs: Protocadherins; ARC: Activity-regulated cytoskeletal; BDNF: Brain-derived neurotrophic factor; MHC II: Major Histocompatibility Complex Class II; IGF2: Insulin growth factor 2; POLD1: Polymerase delta; H19: Long non-coding RNA H19; FOXA1: Fork-head box protein A1; ER: Estrogen receptor; SMAD3/4: Small mother against decapentaplegic 3/4; SP1: Stimulatory protein 1; ND: Not determined; REST: Repressor element 1 (RE1)-silencing transcription; PHF8: PHF finger protein 8; TIP60: Tat-interacting protein of 60 kDa; CREB: cAMP response element binding protein; CBP: CREB binding protein; JMJD3: Jumonji domain-containing protein-3; MeCP2: Methyl-CpG-binding Protein 2; mSin3A: Mammalian switch-independent 3A; HDACs: Histone deacetylases.
